# Systematic review and meta-analysis on the diagnostic accuracy of various detection methods for porcine reproductive and respiratory syndrome virus

**DOI:** 10.1186/s40813-025-00482-1

**Published:** 2026-01-14

**Authors:** Wenxiang Zhang, Tao He, Honghuan Li, Aodi Wu, Xin Li, Qianqian Dong, Jie Chen, Jihai Yi, Jinliang Sheng, Xiangwei Zhao

**Affiliations:** 1 College of Animal Science and Technology, Shihezi University, Shihezi, 832003 China; 2 State Key Laboratory of Digital Medical Engineering, School of Biological Science & Medical Engineering, Southeast University, Nanjing, 210096 China

**Keywords:** PRRSV, Laboratory detection, Meta-analysis, Pigs, Systematic review

## Abstract

**Background:**

Currently, many detection methods for porcine reproductive and respiratory syndrome virus have been developed, However, the optimal laboratory diagnostic method remains controversial. To evaluate the diagnostic accuracy of PRRSV detection methods based on systematic reviews and meta-analyses, and to determine the optimal strategy for laboratory detection of PRRSV.

**Method:**

Articles published between 1 January 2015 and 1 January 2025 were retrieved from multiple databases. Based on different detection methods, the articles were divided into three categories: traditional immunological techniques, molecular amplification techniques, and convergent diagnostic technologies. The sensitivity and specificity of each study were calculated. Diagnostic accuracy was assessed using threshold value definitions, ROC curve analysis, and statistical methods. Meta-analysis was performed using a random-effects model and pooled SROC curves. Stratified analysis and meta-regression were used to address effect size variability caused by differences in detection targets, tissue samples tested, and control trial designs.

**Results:**

A total of 55 articles on traditional immunological techniques (involving 17,359 samples), 90 articles on molecular amplification techniques (involving 21,362 samples), and 14 articles on convergent diagnostic technologies (involving 1,289 samples) were included in the meta-analysis. In the 55 studies on traditional immunological techniques, the overall sensitivity was 0.93–0.94 (95% CI), and the overall specificity was 0.92 (95% CI). The area under the ROC curve (AUC) was 0.9686, with an overall diagnostic odds ratio of 115.23 (95% CI 71.38-186.01). In 90 studies on molecular amplification techniques, the overall sensitivity was 0.97 (95% CI 0.96–0.97), and the overall specificity was 0.99 (95% CI 0.99–0.99). The AUC was 0.9951, with an overall diagnostic odds ratio of 1540 (95% CI 883.97-2684.10). In 14 studies on convergent technologies, the overall sensitivity was 0.95 (95% CI 0.92–0.96), and the overall specificity was 0.98 (95% CI 0.96–0.99). The AUC was 0.9910, with an overall diagnostic odds ratio of 503.74 (95% CI 152.78-1660.88).

**Conclusion:**

The systematic review and meta-analysis results indicate that traditional immunological techniques, molecular amplification techniques, and convergent diagnostic technologies all exhibit high sensitivity and specificity. Among the three technological platforms, molecular amplification techniques consistently yielded the highest point estimates for sensitivity, specificity, and AUC, along with a markedly higher diagnostic odds ratio.

**Supplementary Information:**

The online version contains supplementary material available at 10.1186/s40813-025-00482-1.

## Introduction

Porcine Reproductive and Respiratory Syndrome Virus (PRRSV) is an enveloped, positive-sense single-stranded RNA virus belonging to the family *Arteriviridae* within the order *Nidovirales* [1]. The virus primarily infects porcine macrophages, causing severe reproductive and respiratory diseases [2]. PRRSV is classified into two distinct species: PRRSV-1 (European genotype) and PRRSV-2 (North American genotype), which share only about 50–70% nucleotide sequence identity [3]. Transmission occurs mainly through direct contact with infected pigs and their secretions, but the virus can also spread via aerosol over short distances and through contaminated fomites, contributing to its rapid dissemination among farms [4–7].

Its clinical features include reproductive disorders in sows, high mortality rates (ranging from 60% to 100%) in piglets, and severe respiratory diseases in pigs of all ages [8]. Since the discovery of the Blue Ear Virus in 1991 [9], PRRSV has become endemic in 70 countries worldwide and is one of the most destructive pathogens in the pig farming industry, imposing huge economic losses on the global pig sector. The seroprevalence rate of pigs in North America exceeds 80%, resulting in annual economic losses of up to US$664 million [10]. Recent epidemiological surveys in China show that the detection rate in large-scale pig farms is as high as 32.1%, with 73.6% of cases caused by wild-type strains. The infection rate among suckling piglets reaches 58.9%. During outbreaks, the abortion rate among sows can reach 40%, and mortality rates among piglets in affected herds have been reported to range from 60% to as high as 100% [11].

PRRSV has a wide range of strains, high recombination and mutation rates, and limited vaccine control. The ongoing threat posed by this virus stems from its complex typing and rapid evolution—European type and North American type, with antigenic differences between the two exceeding 40% [11]. Genotype 2 virus is further divided into nine sublineages (Lineages 1 to 9). Recombination and mutation drive the rapid evolution of PRRSV, with an annual mutation rate as high as 1.55 × 10⁻³ substitutions/site/year [12], Notably, the Chinese prevalent strain HP-PRRSV JXA1 frequently recombines with North American NADC30-like/NADC34-like strains. The mutation rate of the ORF5 gene is particularly high, with as many as 78 mutation sites [13]. This genetic diversity directly weakens the effectiveness of vaccine control, resulting in insufficient cross-protection and no cross-protection between genotypes. The neutralising titre of heterologous strains decreases, while Ingelvac PRRS MLV (North American vaccine) reduces the neutralising antibody titre of Chinese Lineage 1 (NADC30-like) strains by 5- to 8-fold [14]. European-type vaccines (based on the Lena strain) do not provide cross-protection against North American wild-type viruses. The SD2020 strain containing the JXA1-R fragment has confirmed the recombination of the vaccine strain and the wild-type strain, exacerbating the complexity of the epidemic [15]. The high variability of PRRSV thus makes its detection particularly important.

In this context, PRRSV is one of many diseases that do not produce specific clinical signs, so accurate detection or differential diagnosis must rely on laboratory testing. To improve diagnostic accuracy, various testing methods have been developed [16]. Over the past three decades (since its discovery in 1991), PRRSV detection methods have advanced rapidly, primarily relying on three major technical categories: Traditional immunological techniques (enzyme-linked immunosorbent assay (ELISA), fluorescent microsphere immunoassay, immunoblotting, etc.) [17–19], Molecular amplification techniques (reverse transcription-polymerase chain reaction (RT-PCR), quantitative reverse transcriptase polymerase chain reaction (qRT-PCR), reverse transcription loop-mediated isothermal amplification (RT-LAMP), etc.) [20–22], Convergent diagnostic technologies (RT-LAMP combined with vertical flow visualization test strips (RT-LAMP-VF), microbead suspension array integrated with one-step asymmetric multiplex RT-PCR, etc.) [23–25]. All three platforms exhibit satisfactory diagnostic performance and play a crucial role in PRRSV prevention, control, and eradication [26]. Although these technologies continue to evolve and show high diagnostic potential, study results are influenced by factors such as sample types, detection targets, control trial designs, and technical platforms. It remains unclear which method demonstrates optimal performance in core diagnostic metrics (e.g., sensitivity and specificity) and is most suitable as a routine or primary laboratory test.

Furthermore, no existing studies have systematically analysed and synthesised the accuracy of these diverse detection methods. Therefore, in this systematic review, we analysed and discussed literature on PRRSV detection from the past decade, pooling diagnostic accuracy metrics for traditional immunological techniques, molecular amplification techniques, and convergent diagnostic techniques to evaluate the accuracy of the three major laboratory detection methods, thereby providing theoretical guidance for PRRSV laboratory diagnostics.

## Materials and methods

### Research selection criteria

Inclusion criteria: (1) Articles describing the development of detection methods for porcine reproductive and respiratory syndrome (including multiplex detection). (2) Each established detection method must include control experiments, such as polymerase chain reaction (PCR), reverse transcription-polymerase chain reaction (RT-PCR), reverse transcription-real-time fluorescent quantitative polymerase chain reaction (RT-qPCR), and commercialised test kits (IDEXX). (3) Studies with more than 10 detection samples are considered eligible. (4) The study must provide the absolute number of diagnostic accuracy tests using a 2 × 2 table, and quality assessment has been manually conducted during the article inclusion process. (5) The article is an original article.

Exclusion criteria: (1) Reviews, editorials, patents, and conference papers were excluded. (2) Duplicate publications were excluded. (3) Studies that did not match the research content were excluded. (4) Studies that established detection methods without control trials were excluded (such as PCR, RT-PCR, RT-qPCR, or commercial ELISA kits). (5) Studies that established detection methods but could not provide complete data for the four groups of true positive (TP), false positive (FP), true negative (TN), and false negative (FN) were excluded.

Some scientific articles reported evaluations of multiple tests for controlled trials. In these cases, each comparison (e.g., using different types of tests) was considered separately. Therefore, the number of studies that needed to be analysed exceeded the number of published papers.

### Study selection and data extraction

Conduct literature searches according to the preferred reporting items for systematic reviews and meta-analyses (PRISMA) updated guidelines [27], A search was conducted in the databases PubMed, Web of Science, and China National Knowledge Infrastructure (CNKI) for articles published between 2015 and 2025 without language restrictions. The following keywords or titles were used: (‘Porcine Reproductive and Respiratory Syndrome,’ ‘Blue Ear Disease,’ and ‘Detection’), and the search was expanded using MeSH subject headings, The search strategy was (Blue-Eared Pig Disease or Blue Eared Pig Disease or Pig Disease, Blue-Eared or Mystery Swine Disease or Swine Disease, Mystery or Porcine Epidemic Abortion and Respiratory Syndrome or PRRS or Swine Infertility and Respiratory Syndrome) and (detection OR inspection OR testing).

All potential studies were assessed using a standardised form (Tables S1, S2 and S3) and a double-blind screening process. Conflicts were resolved through discussion until consensus was reached. The following information was extracted from each study: first author’s name, year of publication, type of detection technology, detection target, sample type, criteria used for selecting controls, and the number of true positives, false positives, true negatives, and false negatives.

### Data analysis and statistical methods

The meta-analysis was performed using Meta-Disc 1.4 software (http://www.hrc.es/investigacion/metadisc_en.htm) [28]. Diagnostic accuracy measures, including sensitivity, specificity, positive and negative likelihood ratios (LR+, LR–), and the diagnostic odds ratio (DOR), along with their 95% confidence intervals (CIs), were calculated for each detection method.

A random-effects model (Der Simonian and Laird method) was used for all pooled estimates to incorporate between-study heterogeneity (τ²), with inverse variance weighting applied for meta-analysis. Sensitivity and specificity were pooled after logit transformation, while the diagnostic odds ratio (DOR) and likelihood ratios (LR+/LR–) were pooled on the natural logarithmic scale. Heterogeneity was assessed using the Cochran Q statistic (with *P* < 0.10 indicating significance) and the I² statistic, where I² values of 25%, 50%, and 75% were considered to represent low, moderate, and high heterogeneity, respectively; I² > 50% indicated substantial heterogeneity [29, 30]. he summary receiver operating characteristic (SROC) curve was generated using the Moses-Shapiro-Littenberg model, which fits a weighted least squares regression between logit(sensitivity) and logit(1 − specificity). The area under the curve (AUC) was used as a global measure of diagnostic accuracy.

To explore sources of heterogeneity, subgroup analyses were conducted based on variables such as control trials, test samples, and test targets. Furthermore, univariable meta-regression analyses were performed using the weighted least squares method within Meta-Disc. The natural logarithm of each study’s DOR (log(DOR)) was regressed against predefined covariates, with the weight for each study being the inverse of the variance of its log(DOR). The results are expressed as the relative diagnostic odds ratio (RDOR) with a 95% CI, which estimates the ratio of DORs between different levels of a categorical covariate or per unit increase in a continuous covariate [31]. All forest plots, stratified analyses, and meta-regression analyses were generated using the software.

Additionally, Egger’s regression test was used to assess funnel plot asymmetry, i.e., by examining the association between effect sizes and standard errors. The generation of funnel plots was conducted using the “metafor” package in R software (Version 4.5.0) [32].

## Result

### Research selection

**Fig. 1:**
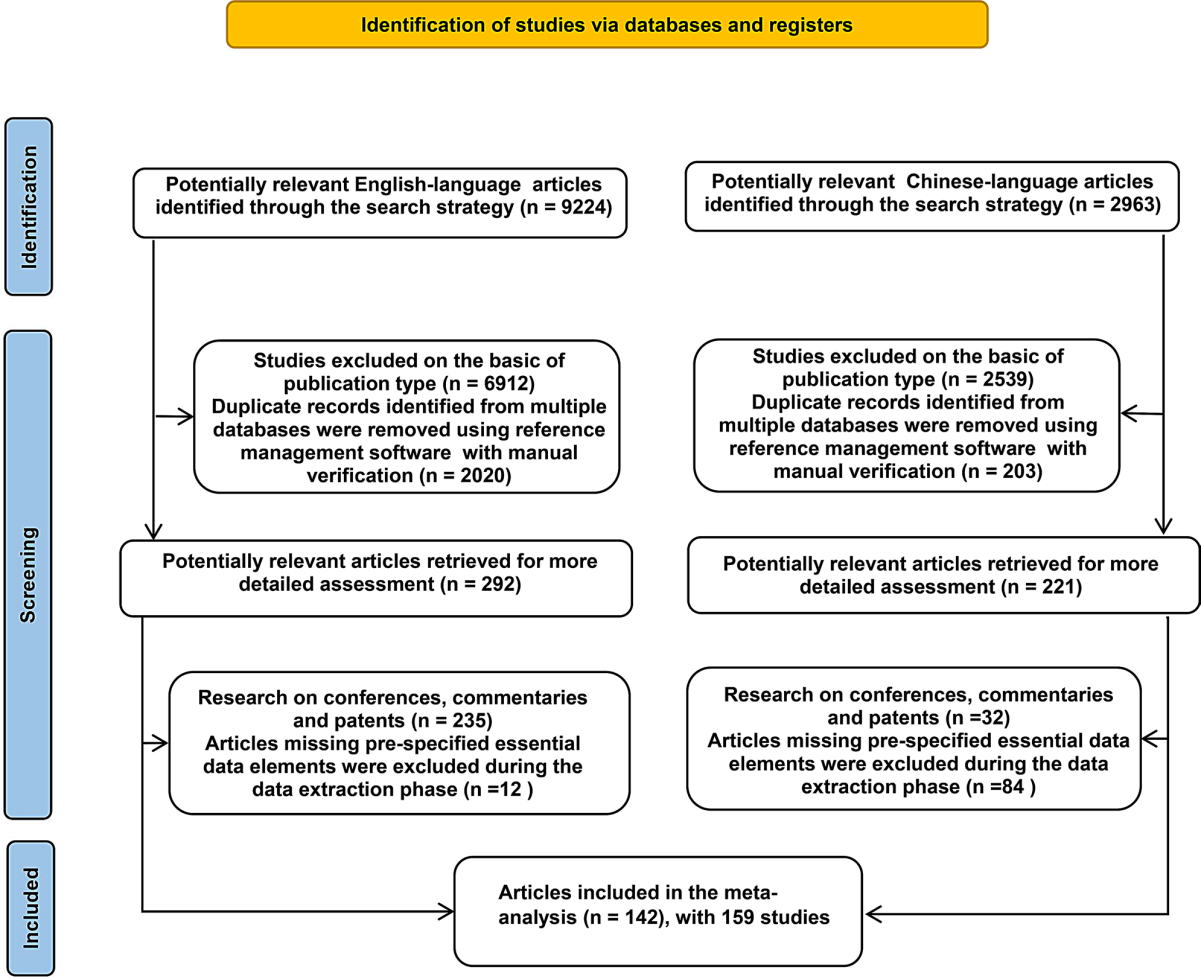
PRISMA 2020 Study selection flow chart

A literature search identified 9,224 English-language articles and 2,963 Chinese-language articles related to PRRSV from 2015 to 2025. A total of 100 Chinese-language articles and 42 English-language articles were included, involving 159 studies and 40,010 research samples, which can be analysed further (Fig. 1).

The study covered eight countries, with most of the research conducted in China (*n* = 128), The remaining studies were conducted in South Korea (*n* = 6), the Czech Republic (*n* = 2), Japan (*n* = 2), Vietnam (*n* = 1), United States (*n* = 1), India (*n* = 1), and Canada (*n* = 1).

### Accuracy determination

#### Traditional immunological technology measurement

**Fig. 2:**
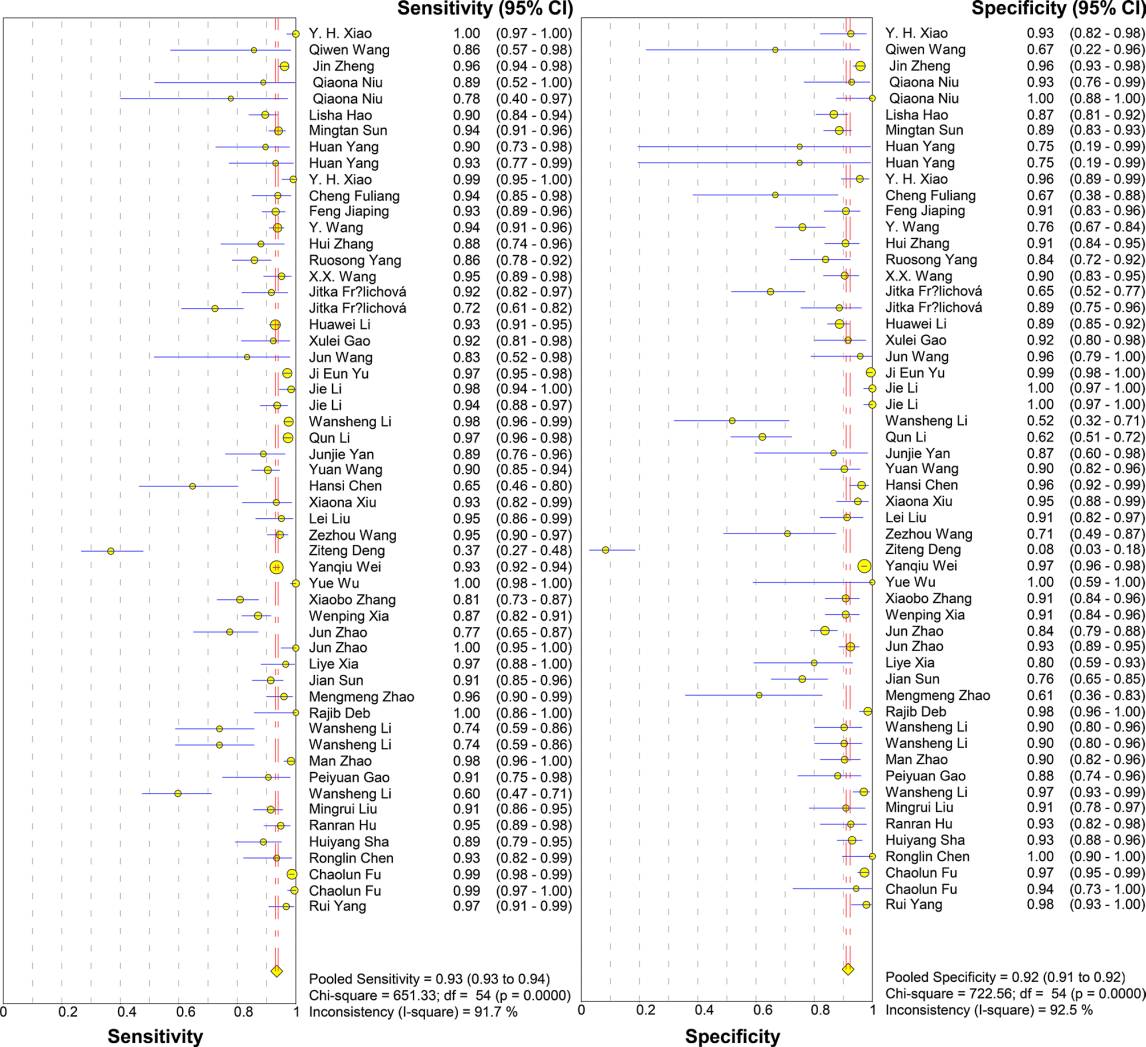
Forest plot of sensitivity and specificity estimates for traditional immunological techniques. ●(golden), Point estimates of sensitivity from each study (proportional to size of the study); -, 95% confidence intervals; ◆(golden), pooled sensitivity and specificity estimates

**Fig. 3:**
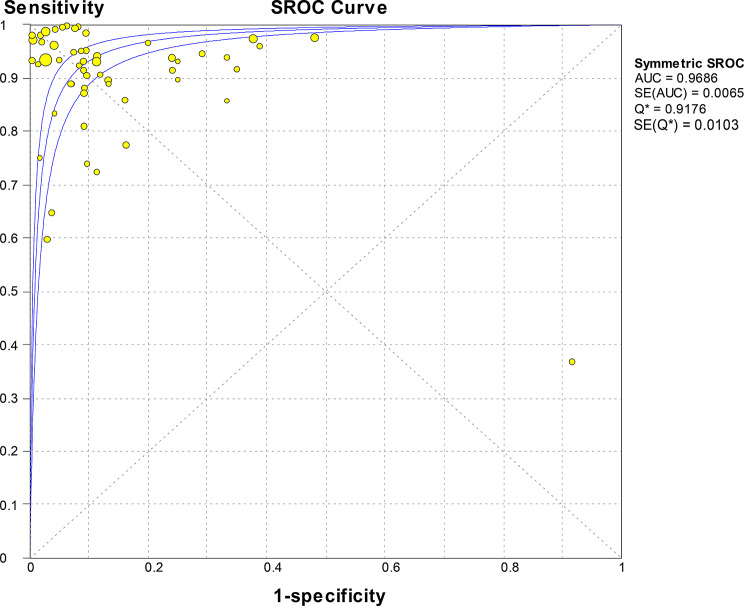
SROC curve analysis for traditional immunological techniques: individual studies are represented as golden dots, with dot size proportional to sample size. The symmetric SROC curve (central line) with its 95% interval confidence (side lines) was created using the overall diagnostic odds ratio of all the studies

Immunological detection techniques including Enzyme-linked immunosorbent assay (ELISA), immunoblotting assay, immunochromatographic strip test, luciferase-coupled antibody capture assay based on a luciferase immunoprecipitation system, immunochromatographic strip test (ICST), lanthanide-based fluorescent immunochromatography (LFICA), protein chip detection technology, fluorescent microsphere immunochromatographic strip assay (FICT) technology, liquid phase chip technology (xMAP), immunochromatographic strip (ICS) detection technology based on latex microspheres, colloidal gold immunochromatography, quantum dot fluorescent microsphere immunochromatographic strip (QDFM ICS), and fluorescent microsphere immunochromatography technology. with a total of 55 studies. A total of 17,359 samples were provided. The meta-analysis conducted using a random-effects model showed that the pooled sensitivity was 0.93 (95% CI 0.93–0.94) with an I² statistic of 91.7%, and the pooled specificity was 0.92 (95% CI: 0.91–0.92) with a corresponding I² statistic of 92.5% (Fig. 2). The LR་

 and LR- were 9.41 (95% CI 6.60-13.41) and 0.09 (95% CI 0.07–0.12), respectively, with a DOR of 115.23 (95% CI 71.38-186.01), indicating high accuracy. SROC curve analysis did not show statistically significant heterogeneity, with a Q value of 0.9176 and SE (Q*) = 0.0103. The AUC was 0.9686 (Fig. 3).

#### Molecular amplification technology measurement

**Fig. 4:**
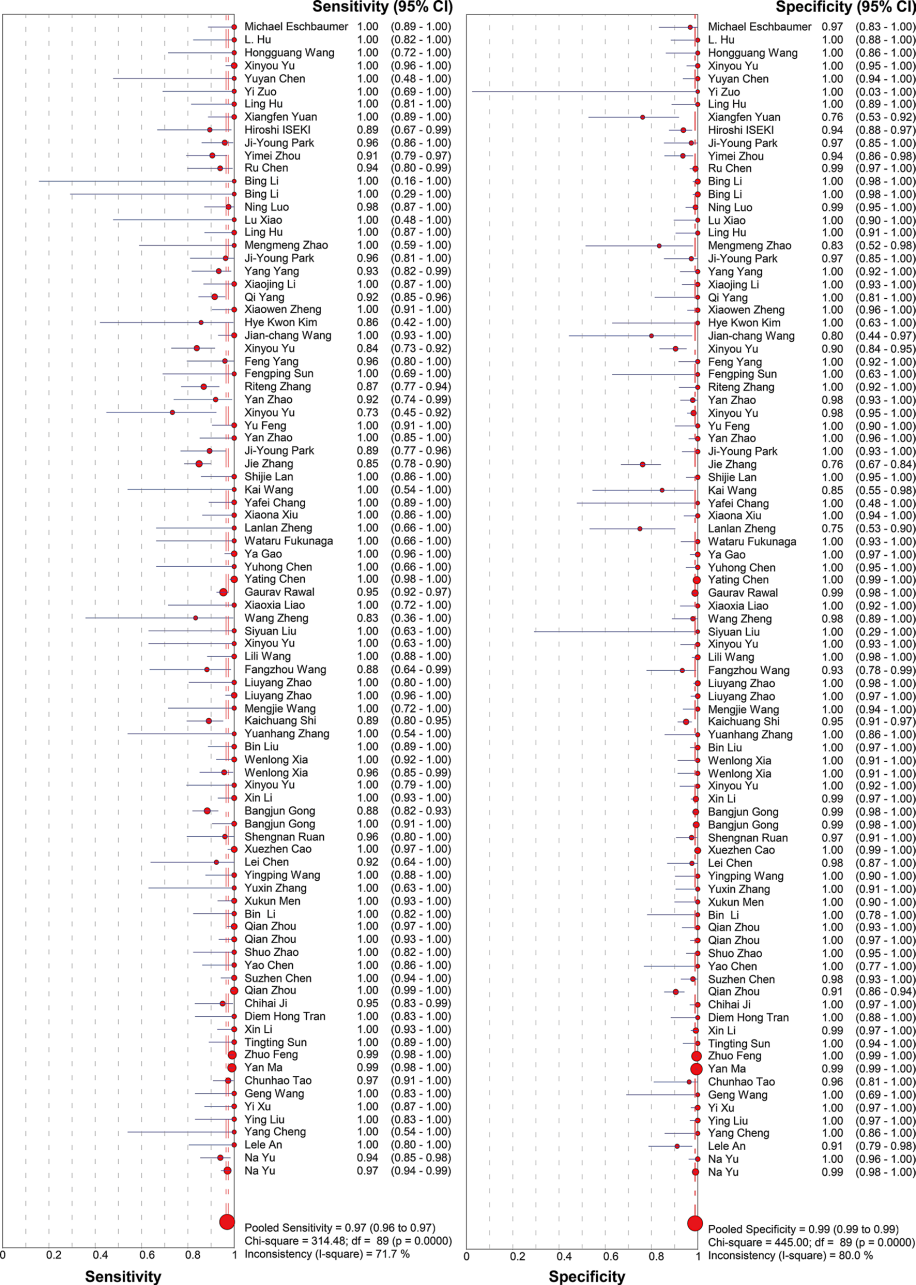
Forest plot for sensitivity and specificity estimates of molecular amplification techniques. ●(red), Point estimates of sensitivity from each study (proportional to size of the study); -, 95% confidence intervals; ◆(red), pooled sensitivity and specificity estimates

**Fig. 5:**
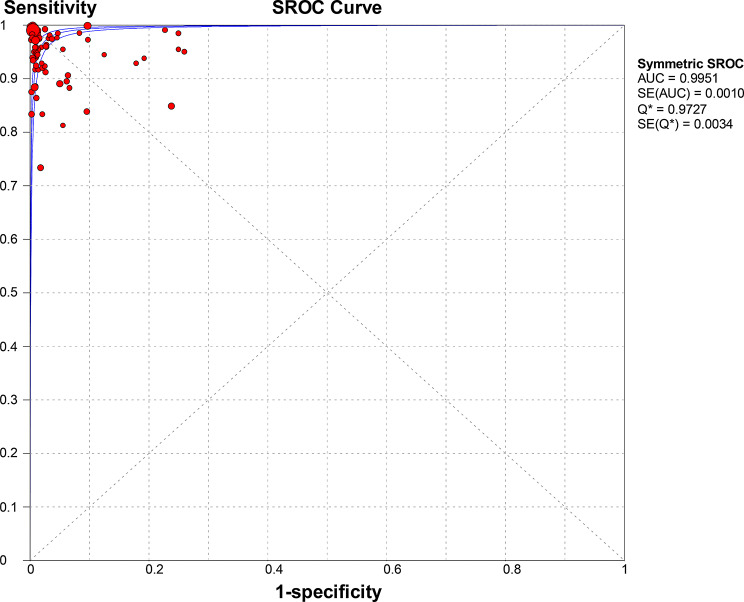
SROC curve analysis for molecular amplification techniques: Individual studies are depicted as red dots., with dot size proportional to sample size. The symmetric SROC curve (central line) with its 95% interval confidence (side lines) was created using the overall diagnostic odds ratio of all the studies

Molecular amplification detection technologies, including: Reverse Transcription Polymerase Chain Reaction(RT-PCR)、Multiplex Polymerase Chain Reaction (mPCR)、Multiplex Reverse Transcription PCR (mRT-PCR)、Duplex Reverse Transcription PCR(Duplex RT-PCR)、Reverse Transcription Loop-mediated Isothermal Amplification (RT-LAMP)、Genome Lab Gene Expression Profiler PCR (GeXP-PCR)、Droplet Digital Polymerase Chain Reaction (ddPCR)、Digital Polymerase Chain Reaction (dPCR)、Reverse Transcription Recombinase Polymerase Amplification (RT-RPA)、Quantitative Real-Time Reverse Transcription (PCR) qRT-PCR、Multiple Annealing and Looping-Based Amplification (MAOPA)、Reverse Transcription Recombinase-Aid Amplification (RT-RAA), isothermal amplification technologies (RT-LAMP, RPA/RAA, etc.), and CRISPR-based detection, were included in a total of 90 studies. A total of 21,362 samples were provided. The meta-analysis conducted using a random-effects model showed that the pooled sensitivity was 0.97 (95% CI 0.96–0.97) with an I² statistic of 71.7%, and the pooled specificity was 0.99 (95% CI 0.99–0.99) with a corresponding I² statistic of 80% (Fig. 4). LR་ and LR- values were 49.88 (95% CI 33.93–73.33) and 0.04 (95% CI 0.03–0.06), respectively. The DOR was 1540 (95% CI 883.97-2684.10), indicating high accuracy. The SROC curve analysis did not show statistically significant heterogeneity, with a Q value of 0.9727, SE (Q*) = 0.0034, and an AUC of 0.9951 (Fig. 5).

#### Convergent diagnostic technology measurement

**Fig. 6:**
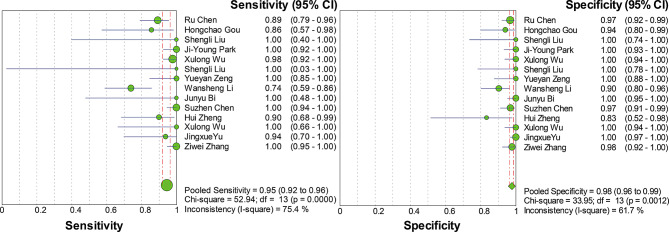
Forest plot for sensitivity and specificity estimates of convergent diagnostic technologies. ● (green), Point estimates of sensitivity from each study (proportional to size of the study); -, 95% confidence intervals; ◆ (green), pooled sensitivity and specificity estimates

**Fig. 7:**
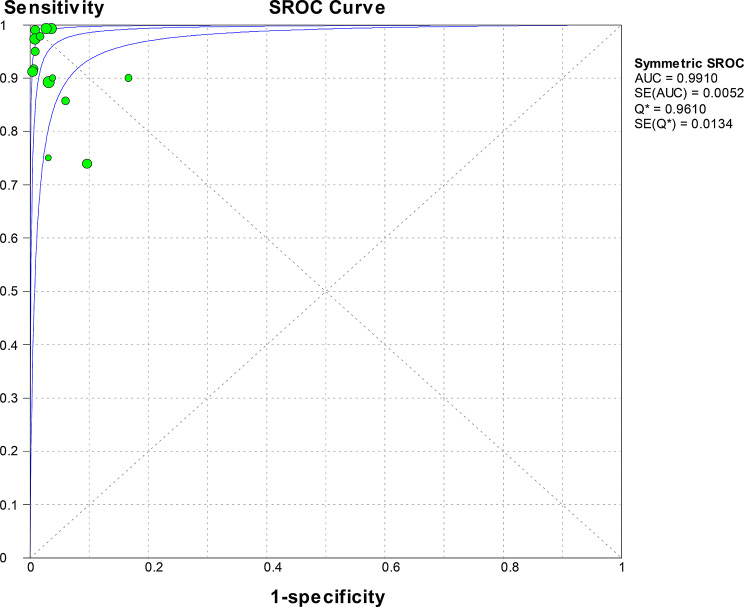
SROC curve analysis for convergent diagnostic technologies: Individual studies are depicted as green dots., with dot size proportional to sample size. The symmetric SROC curve (central line) with its 95% interval confidence (side lines) was created using the overall diagnostic odds ratio of all the studies

Convergent diagnostic technologies, including Based on microbead suspension array technology, combined with one-step asymmetric multiplex reverse transcription polymerase chain reaction (RT-PCR), reverse transcription loop-mediated isothermal amplification combined with vertical flow visualisation test strips (RT-LAMP - VF), multiplex PCR combined with gene chip technology, one-step real-time fluorescent quantitative RT-PCR mediated by peptide nucleic acid (PNA) probes combined with fluorescent melting curve analysis (FMCA) technology, multiplex PCR combined with QIAxcel capillary electrophoresis, recombinase polymerase amplification combined with lateral flow chromatographic technology (RPA-LFD), real-time fluorescent quantitative recombinase polymerase amplification (RT-qRPA), recombinase polymerase amplification - lateral flow chromatographic test strip (RPA-LFD), reverse transcription recombinase-assisted amplification - lateral flow chromatographic technology (RT-RAA-LF), PCR combined with fluorescent microsphere immunochromatography strip technology, liquid-phase chip detection (multiplex PCR combined with microsphere hybridisation technology), CRISPR/Cas12a combined with reverse transcription recombinase-mediated isothermal amplification (RT-RAA) technology, reverse transcription recombinase-assisted amplification-lateral flow chromatographic technology (RT-RAA-LF), and immunochromatographic strip (ICS) combined with PCR. were used in a total of 14 studies, providing 1,289 samples. The meta-analysis conducted using a random-effects model showed that the pooled sensitivity was 0.95 (95% CI 0.92–0.96) with an I² statistic of 75.4%, and the pooled specificity was 0.98 (95% CI 0.96–0.99) with a corresponding I² statistic of 61.7% (Fig. 6). The likelihood ratios LR་ and LR- were 25.44 (95% CI 13.93–46.47) and 0.07 (95% CI 0.03–0.15), respectively. The DOR was 503.74 (95% CI 152.78-1660.88), indicating high accuracy. ROC curve analysis did not show statistically significant heterogeneity, with a Q value of 0.9610, SE (Q*) = 0.0134, and an AUC of 0.9910 (Fig. 7).

### Subgroup analysis and heterogeneity

One of the main causes of heterogeneity in test accuracy studies is the threshold effect, which occurs when differences in sensitivity and specificity are due to the selection of different cut-off values to define positive (or negative) test results [28]. The sensitivity and specificity of the three detection methods varied to varying degrees. This heterogeneity may be due to differences in the detection samples (serum, tissue, and mixed samples) and the types of targets used in each detection method or differences in the control tests. Subgroup analysis was performed to determine the factors associated with heterogeneity (Tables 1, 2 and 3).

**Table 1:** Summary of measures for subgroup analysis comparison in traditional immunological techniques

			Sensitivity		Specificity		
Sub-analysis	Global effect and subgroup analysis restricted	Studies (n)	Pooled estimate (95% CI)	Q (*P*)	Pooled estimate (95% CI)	Q (*P*)	Diagnostic odds ratio
	Global effect	55	0.935 (0.930–0.939)	< 0.001	0.917 (0.910–0.923)	< 0.001	115.23 (71.377–186.01)
Types of test samples							
	Serum samples	52	0.938 (0.933–0.942)	< 0.001	0.913 (0.906–0.920)	< 0.001	116.76 (71.006-192.00)
	Fixed samples	3	0.711 (0.629–0.784)	< 0.001	0.967 (0.945–0.982)	< 0.001	81.188 (CI 15.350-429.43)
Detection targets							
	Structural proteins	32	0.925 (0.917–0.932)	< 0.001	0.897 (0.886–0.907)	< 0.001	94.825 (46.509–193.33)
	Non-Structural proteins	18	0.947 (0.941–0.954)	< 0.001	0.944 (0.935–0.952)	< 0.001	192.38 (97.282–380.45)
Controlled trial							
	RT-PCR	2	0.829 (0.720–0.908)	< 0.001	0.965 (0.934–0.984)	< 0.001	214.46 (2.217-20745.7)
	IDEXX	52	0.938 (0.933–0.942)	< 0.001	0.913 (0.906–0.920)	< 0.001	116.76 (71.006-192.00)

CI, Confidence interval

**Table 2:** Summary of measures for subgroup analysis comparison in molecular amplification techniques

			Sensitivity		Specificity		
Sub-analysis	Global effect and subgroup analysis restricted	Studies (n)	Pooled estimate (95% CI)	Q (*P*)	Pooled estimate (95% CI)	Q (*P*)	Diagnostic odds ratio
	Global effect	90	0.970 (0.965–0.974)	< 0.001	0.989 (0.987–0.990)	< 0.001	1540.3 (883.97-2684.01)
Types of test samples							
	Serum samples	50	0.978 (0.972–0.984)	< 0.001	0.991 (0.989–0.993)	< 0.001	1519.8 (720.27-3206.6)
	Tissue samples	18	0.968 (0.957–0.977)	< 0.001	0.986 (0.981–0.990)	< 0.001	1855.0 (852.39-4036.8)
	Mixed samples	21	0.951 (0.937–0.963)	< 0.001	0.980 (0.973–0.985)	< 0.001	1229.9 (356.78-4240.1)
Detection targets							
	Structural proteins	15	0.966 (0.950–0.977)	< 0.001	0.994 (0.991–0.997)	0.051	2196.7 (995.09-4849.2)
	Non-Structural proteins	54	0.976 (0.970–0.981)	< 0.001	0.960 (0.988–0.992)	< 0.001	1988.8 (867.13-4561.4)
Controlled trial							
	PCR	32	0.964 (0.951–0.973)	< 0.001	0.979 (0.972–0.984)	< 0.001	1164.9 (405.21-3349.9)
	RT-PCR	32	0.959 (0.949–0.968)	< 0.001	0.987 (0.984–0.991)	< 0.001	1247.8 (560.46–2778.0)
	RT-qPCR	26	0.981 (0.975–0.987)	< 0.001	0.992 (0.990–0.994)	< 0.001	2731.5 (1356.7-5499.6)

CI, Confidence interval

**Table 3:** Summary of measures for subgroup analysis comparison in convergent diagnostic technologies

			Sensitivity		specificity		
Sub-analysis	Global effect and subgroup analysis restricted	Studie (n)	Pooled estimate (95% CI)	Q (*P*)	Pooled estimate (95% CI)	Q (*P*)	Diagnostic odds ratio
	Global effect	14	0.945 (CI, 0.920–0.964)	0.001	0.977 (CI, 0.964–0.986)	< 0.001	503.74 (95% CI 152.78-1660.9)
Types of test samples							
	Serum samples	5	0.989 (CI, 0.960–0.999)	0.062	0.977 (CI, 0.953–0.991)	0.044	1001.2 (95% CI 115.03-8713.7)
	Tissue samples	7	0.973 (CI, 0.937–0.991)	0.267	0.994 (CI, 0.978–0.999)	0.156	736.48 (95% CI 161.23-3364.1)
	Mixed samples	2	0.829 (CI, 0.746–0.894)	0.036	0.946 (CI, 0.903–0.974)	0.076	78.913 (95% CI 8.786–708.79)
Detection targets							
	Structural proteins	7	0.933 (CI, 0.891–0.963)	< 0.001	0.969 (CI, 0.948–0.984)	0.015	461.01 (95% CI 65.810-3229.4)
	Non-Structural proteins	2	0.974 (CI, 0.862–0.999)	0.184	1.000 (CI, 0.973- 1.000)	1	2327.4 (95% CI 189.25-28624.2)
Controlled trial							
	PCR	8	0.946 (CI, 0.911–0.970)	< 0.001	0.967 (CI, 0.944–0.982)	0.011	439.29 (95% CI 63.702–3029.4)
	RT-PCR	3	0.969 (CI, 0.922–0.991)	0.089	0.983 (CI, 0.940–0.998)	0.076	742.84 (95% CI 49.135-11230.6)
	RT-qPCR	3	0.907 (CI, 0.825–0.959)	0.541	0.987 (CI, 0.967–0.996)	0.027	395.98 (95% CI 117.72–1332.0)

CI, Confidence interval

#### Immunological testing technology stratification analysis

Based on two different types of test samples, serum was the sample of choice in 52 studies. The sensitivity (0.938, 95% CI 0.933–0.942) was significantly higher than that of mixed samples (*n* = 3) (0.711, 95% CI 0.629–0.784). and the DOR 95% CI (15.350-429.43) was wide, which may be due to the small sample size and significant differences among the three studies. The specificity of serum samples (0.913, 95% CI 0.906–0.920) was lower than that of mixed samples (0.967, 95% CI 0.945–0.982). The use of the test samples did not significantly reduce heterogeneity.

Subgroup analysis was divided into structural proteins (*n* = 32 GP5, M, N) and non-structural proteins (*n* = 18 Nsp1 α, Nsp2, Nsp4, Nsp7, Nsp9, Nsp10, Nsp12). The sensitivity (0.925, 95% CI 0.917–0.932) and specificity (0.897, 95% CI 0.886–0.907) of the subgroup using structural proteins as detection targets were both lower than those of the subgroup using non-structural proteins as detection targets sensitivity (0.947, 95% CI 0.941–0.954) and specificity (0.944, 95% CI 0.935–0.952) of the subgroup using non-structural proteins as detection targets, indicating that the choice of detection targets significantly affects diagnostic performance, particularly specificity. However, these two stratified studies still exhibited significant heterogeneity.

There were two control trials in the subgroup. The sensitivity of the IDEXX commercial kit (*n* = 52) as the control trial (0.938, 95% CI 0.933–0.942) was significantly higher than that of RT-PCR (*n* = 2) as the control trial (0.829, 95% CI 0.720–0.908). while the specificity results were the opposite. However, the DOR (214.46) point estimate for the RT-PCR group was higher than that for the IDEXX group (116.76), but its confidence interval (2.217-20745.7) was unusually wide. Such results may be due to the extremely small number of subgroup studies using RT-PCR as the control test. Overall, the stratified studies exhibited significant heterogeneity.

#### Stratified analysis of molecular amplification technology

Subgroup analyses based on the three different types of detection samples showed that serum was used as the detection sample in 50 studies. The sensitivity and specificity values for this subgroup were 0.978 (95% CI 0.972–0.984) and 0.991 (95% CI 0.989–0.993), respectively. When tissue samples (*n* = 18, including lung, lymph nodes, heart, and spleen, etc.) were used for testing, the pooled sensitivity was 0.968 (95% CI 0.957–0.977), and the pooled specificity was 0.986 (95% CI 0.981–0.990). However, when using mixed samples (*n* = 21, including tissue and serum), both sensitivity (0.951, 95% CI 0.937–0.963) and specificity (0.980, 95% CI 0.973–0.985) were lower than in the other two groups. However, all subgroup analyses showed significant heterogeneity (*P* < 0.001).

By detecting different targets, the subgroups were divided into structural proteins (*n* = 15: GP2, GP5, M, N) and non-structural proteins (*n* = 54: Nsp1 α, Nsp2, Nsp7, Nsp9). The sensitivity of the subgroup using structural proteins as detection targets (0.966, 95% CI 0.950–0.977) was lower than that of the subgroup using non-structural proteins as detection targets (0.976, 95% CI 0.970–0.981).

Given that each study used different control tests, subgroup analysis showed that PCR (*n* = 32) had the lowest sensitivity (0.964, 95% CI 0.951–0.973) and specificity (0.979, 95% CI 0.972–0.984) when used as the control test. while using RT-qPCR (*n* = 26) as the control test yielded the highest sensitivity (0.981, CI 0.975–0.987) and specificity (0.992, CI 0.990–0.994). However, heterogeneity among all control tests was significant (*P* < 0.001).

#### Convergent diagnostic technologies stratified analysis

Subgroup analysis was conducted based on sample type, with seven studies involving tissue samples as the test samples. Sensitivity (0.938, 95% CI 0.933–0.942, *P* = 0.267) and specificity (0.994, 95% CI 0.978–0.999, *P* = 0.156) showed no heterogeneity, and the use of tissue samples may be an important factor in reducing heterogeneity. However, the remaining studies using serum (*n* = 5) and mixed samples (*n* = 2) as testing samples still showed significant heterogeneity in sensitivity and specificity.

Subgroup analysis was divided into structural proteins (n = 7 GP2a, M, N) and non-structural proteins (n = 2 Nsp2, 3’-UTR) based on the differences in detection targets. The sensitivity of the subgroup using structural proteins as detection targets (0.933, 95% CI 0.891–0.963) and specificity (0.969, 95% CI 0.948–0.984) were both lower than those of the subgroup using non-structural proteins as detection targets (sensitivity 0.974, 95% CI 0.862–0.999; specificity 1.000, 95% CI 0.973-1.000). The structural protein subgroup analysis still showed significant heterogeneity, but the non-structural protein subgroup analysis did not show significant heterogeneity.

Subgroup PCR (*n* = 8) as a control trial sensitivity (0.946, 95% CI 0.911–0.970) and specificity (0.967, 95% CI 0.944–0.982) were lower than those of RT-PCR (*n* = 2) as the control test (sensitivity: 0.969, 95% CI: 0.922–0.991; specificity: 0.983, 95% CI: 0.940–0.998), However, although the sensitivity of the RT-qPCR group (0.907, 95% CI 0.825–0.959) was lower than that of the other two groups, its heterogeneity was not significant (*P* = 0.541).

When non-structural proteins are used as detection targets in the three types of detection technologies, the specificity and sensitivity of the detection methods are higher than those of structural proteins. When blood samples are used for PRRSV detection, their sensitivity and specificity are higher than those of the other two groups.

### Publication bias assessment

**Fig. 8:**
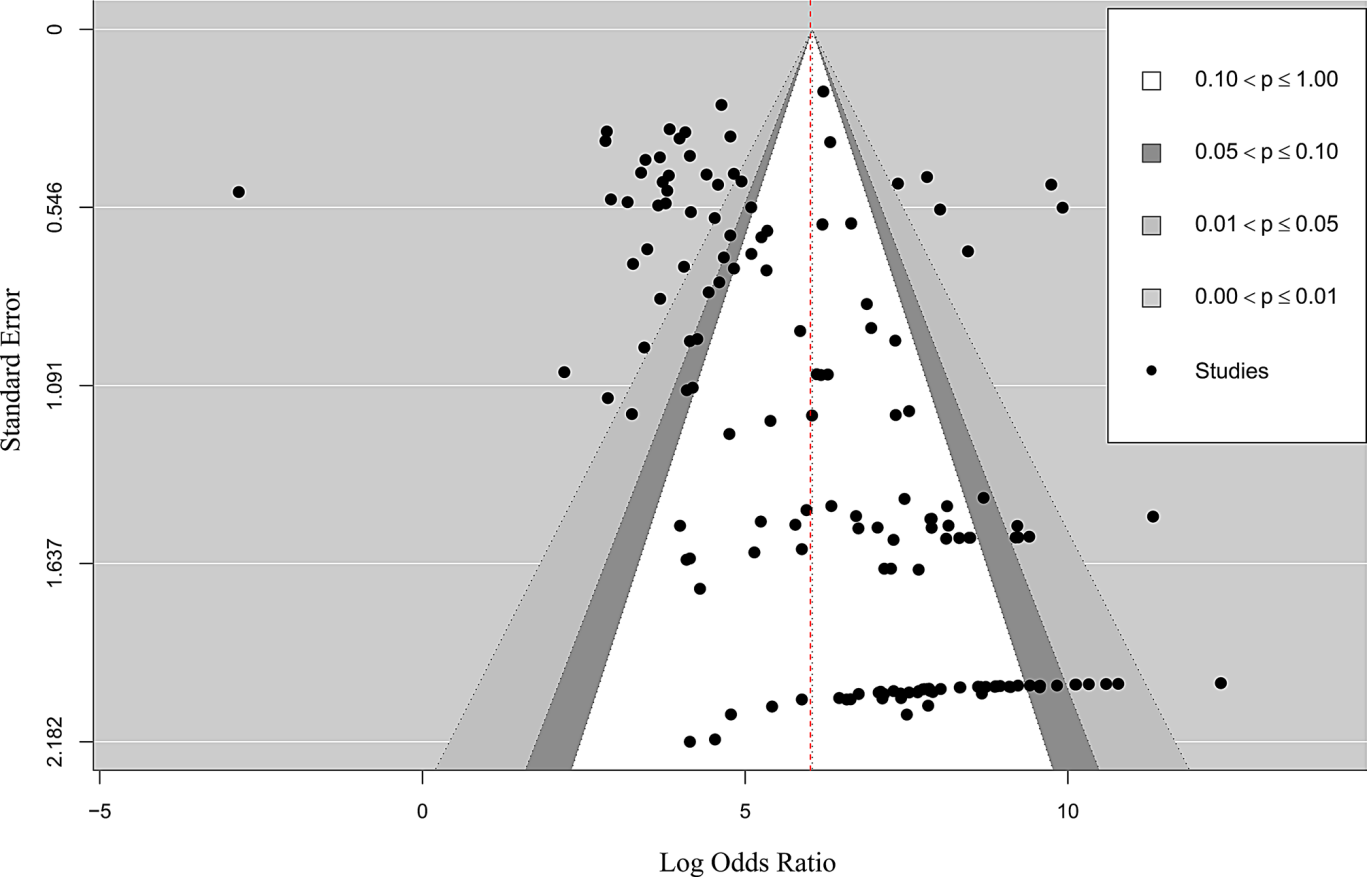
Publication bias

Publication bias was assessed using Egger’s linear regression test and Begg’s rank correlation test. Both tests indicated significant funnel plot asymmetry (Egger’s test: z = 8.83, *p <* 0.001; Begg’s test: Kendall’s τ = 0.22, *p* < 0.001), suggesting the potential presence of publication bias in this meta-analysis (Fig. 8).

### Meta-regression analysis

The stratified analysis results in this review cannot fully explain the significant heterogeneity among individual studies; therefore, a meta-regression meta-analysis was conducted to assess multiple factors. The regression analysis results are RDOR (Tables 4, 5 and 6). In the meta-regression analysis of traditional immunological techniques and molecular amplification techniques, three covariates were included: detection target, region, and publication year. The RDOR differences between studies grouped by detection target and region were not significant, while the RDOR differences between studies grouped by publication year were significant. In cross-platform combination technologies, since the data were too scattered to perform regression analysis, only the detection target and publication year were included as covariates. The differences in RDOR for both were not significant, indicating that the detection target and region do not substantially affect diagnostic accuracy.

**Table 4:** Meta-Regression analysis to identify sources of heterogeneity in traditional immunological techniques

Covariate	Coefficient	*P* value	RDOR	95% CI
Intercept	3.941	< 0.0001	-	-
Threshold (S)	-0.102	0.5932	-	-
Detection target	0.171	0.2040	1.19	(0.91–1.55)
Region	0.142	0.8176	1.15	(0.34–3.96)
Publication date (year)	0.050	0.6290	1.05	(0.85–1.30)

RDOR, Relative diagnostic odds ratio; CI, confidence interval

Intercept = constant in the model

S, Indicator of threshold (logit true positive rate ་ logit false positive rate)

Covariates tested in the meta-regression: Detection target (structural vs. non-structural proteins), Region (as a categorical variable), Publication year (as a continuous variable)

**Table 5:** Meta-Regression analysis to identify sources of heterogeneity in molecular amplification techniques

Covariate	Coefficient	*P* value	RDOR	95% CI
Intercept	6.632	< 0.001	-	-
Threshold (S)	-0.206	0.323	-	-
Detection target	-0.175	0.3423	0.84	(0.58–1.21)
Region	-0.258	0.4856	0.77	(0.37–1.61)
Publication date (year)	-0.231	0.0103	1.26	(1.06–1.50)

RDOR, Relative diagnostic odds ratio; CI, confidence interval

Intercept = constant in the model

S, Indicator of threshold (logit true positive rate ་ logit false positive rate)

Covariates tested in the meta-regression: Detection target (structural vs. non-structural proteins), Region (as a categorical variable), Publication year (as a continuous variable)

**Table 6:** Meta-Regression analysis to identify sources of heterogeneity in convergent diagnostic technologies

Covariate	Coefficient	*P* value	RDOR	95% CI
Intercept	4.034	0.0596	-	-
Threshold (S)	0.441	0.5669	-	-
Detection target	0.136	0.5498	1.15	(0.28–4.71)
Publication date (year)	0.936	0.6542	2.55	(0.47–13.71)

RDOR, Relative diagnostic odds ratio; CI, confidence interval

Intercept = constant in the model

S, Indicator of threshold (logit true positive rate ་ logit false positive rate)

Covariates tested in the meta-regression: Detection target (structural vs. non-structural proteins), Region (as a categorical variable), Publication year (as a continuous variable)

## Discussion

This meta-analysis adhered to the standard protocol for systematic reviews, incorporating studies published in different languages. Two reviewers independently screened articles and extracted data, employing various methods, including SROC analysis, heterogeneity exploration, and meta-regression. The meta-analysis was based on 150 published studies evaluating PRRSV detection, categorising the studies into three groups: traditional immunological techniques, molecular amplification detection techniques, and convergent diagnostic technologies. TP, FP, TN and FN were extracted and data were summarised, showing area under the SROC curve areas of 0.9682, 0.9951, and 0.9910 for the three groups, respectively. All three methods exhibited high diagnostic performance, with molecular amplification techniques demonstrating the highest diagnostic value. Molecular amplification techniques exhibit very high diagnostic accuracy. Within this category, techniques such as RT-LAMP are particularly notable; published studies highlight its advantages of rapidity, results visible to the naked eye, and no cross-reactivity with bacteria or other viruses [33], making it a strong candidate for primary diagnosis. However, the pooled analysis in this study does not provide statistical evidence to rank individual molecular techniques against each other.

Multiple linkage probes amplification (MLPA) utilises probes hybridised to sample DNA with a single pair of PCR primers, allowing semi-quantitative analysis of up to 60 target genes in a single reaction. This method effectively addresses PRRSV genetic variability and reduces cross-reactivity with other viruses [34]. For HP-PRRSV control, the attenuated vaccine (HP-PRRSV JXA1-R) is used; however, mixed PRRSV infections have increased. A developed detection method for HP-PRRSV JXA1-R, HP-PRRSV, and C-PRRSV—with a sensitivity of 24 copies/µL and 100% consistency with sequencing results—exhibits high specificity, sensitivity, and reliability for distinguishing between strains, serving as an excellent tool for rapid PRRSV identification [35].

Substantial heterogeneity in overall sensitivity and specificity may compromise the effectiveness of clinical testing. To explore this heterogeneity, we performed a subgroup analysis, identifying the primary sources of variation as: (1) Tested sample types; (2) Detection targets; (3) selection of different control assays. Subgroup analyses showed significant heterogeneity in most cases. The presence of obvious heterogeneity in the results cannot be fully used to explain the sources of different variations. Therefore, we consider that variability in the study design is also one of the sources of heterogeneity. Due to the lack of available information, statistical characteristics (age, gender), disease severity, incidence rate, and regional and feeding methods may be unquantifiable variables that contribute to heterogeneity in this study and warrant further investigation [31].

The detection target is a critical parameter to consider when establishing a detection method, and an excellent detection method is inseparable from the detection target. In our meta-analysis, we found that the specificity and sensitivity were higher when non-structural proteins were used as detection targets compared to other targets. In the stratified analysis, the use of non-structural proteins as detection targets in cross-platform technologies did not result in significant heterogeneity, suggesting that the detection target may be one of the sources of reduced heterogeneity. This finding is attributed to the biological characteristics, evolutionary conservation, and clinical infection dynamics of PRRSV. Structural proteins primarily exist on the surface of viral particles and are expressed during viral assembly in the middle to late stages of infection, making detection possible only after 24 h of infection [36, 37], However, non-structural proteins are only present in infected cells and are expressed early in viral replication, becoming detectable 6 h after infection [38], Furthermore, targeting the conserved regions of Nsp2/Nsp5 (such as the protease activity domain) can avoid the highly variable regions of GP5/GP2a, reducing the risk of primer/antibody ineffectiveness due to strain variation and improving the broad spectrum and specificity of molecular detection [36, 39].

Stratified analysis indicated that the use of different test samples (serum vs. tissue) significantly influenced test results and heterogeneity, a difference largely attributable to the distinct biological behavior of PRRSV. The kinetics of viral infection provide a clear rationale for the observed diagnostic performance: serum viremia typically clears within 30 days, with a peak RT-PCR positivity rate between 3 and 5 days and a significant decline after 21 days [40, 41]. Serum samples exhibit high sensitivity during acute-phase detection, but their results vary with the timing of sampling. In contrast, the reduced heterogeneity and robust performance of tissue samples, such as lymph nodes and tonsils, stem from the prolonged persistence of the virus at these sites, up to 135–180 days [40]. During the acute phase, while serum viral load may be low, viral replication in lung tissue reaches high levels; during persistent infection, the virus is only intermittently detectable in serum, whereas lymphoid tissues consistently harbor the virus, acting as a ‘viral reservoir’ that evades systemic immunity, this underlies the diagnostic reliability of tissue sampling across infection stages [40, 42, 43]. Furthermore, viral detection in the pulmonary lymph nodes of seronegative pigs as late as 60 days post-infection underscores the critical advantage of tissue sampling in detecting latent or persistent infections [42]. From a virological perspective, the organization of viral RNA in tissues also facilitates the acquisition of full-length genomes, as evidenced by the exclusive detection of complete NADC34-like recombinant strains in lung tissue. At the same time, the presence of inhibitors such as haemoglobin and lipoproteins in serum may further compromise diagnostic consistency in serum-based assays [44, 45]. Collectively, the biological features of PRRSV, such as its tissue tropism, persistence, and differences in sample matrix composition, directly explain the differential diagnostic performance found in the meta-analysis. These findings thereby highlight the critical importance of selecting samples according to the infection stage and diagnostic goals.

Under controlled laboratory conditions, molecular amplification techniques provide the highest diagnostic accuracy. However, translating these findings into broad field application strategies requires a balanced consideration of cost-effectiveness, operational feasibility, and real-world conditions. For example, while real-time quantitative PCR (RT-qPCR) offers high sensitivity, it relies on expensive instrumentation and specialized technicians, making it more suitable for large reference laboratories conducting precise diagnosis and strain typing. In contrast, techniques such as reverse transcription loop-mediated isothermal amplification (RT-LAMP), with their low equipment requirements and rapid detection speed, show significant advantages for primary diagnostic settings or on-site use at farm gates where quick decision-making is needed. For purposes such as serological monitoring and large-scale screening, well-validated ELISA kits remain a highly practical option due to their low cost and operational simplicity. Therefore, the optimal detection strategy should not be based solely on analytical performance but should instead involve a decision-making process that considers specific diagnostic objectives, available resources, and required turnaround time. An effective PRRSV control strategy requires the differential selection of detection methods tailored to specific scenarios.

Publication year represents a common source of heterogeneity in meta-analysis, a pattern observed in PRRSV detection due to continuous technological advances. Key developments include the standardization of reagents through commercial kits, the establishment of unified protocols for nucleic acid extraction and amplification, and improved instrumental sensitivity, with consistent detection limits as low as 0.31 ng/mL achieved across various platforms [46]. Concurrently, a deepening understanding of viral genetics has further refined the design of detection methods.

For traditional immunological techniques, false positives may result from cross-reactivity with antibodies against other porcine pathogens or non-specific binding [47, 48], while false negatives can occur during the early seroconversion window or due to antigenic variation in emerging viral strains that evade antibody detection [48]. In molecular amplification techniques, false positives are most frequently associated with amplicon contamination during laboratory processing or, in some cases, primer/probe mismatches resulting from the high genetic variability of PRRSV [49–51]. Although convergent technologies are designed to integrate the advantages of multiple platforms, they may still inherit the limitations of their constituent immunological or molecular components. The use of a random-effects model incorporated heterogeneity arising from such technical variations, and subgroup analyses and meta-regression identified specific factors influencing accuracy. Furthermore, the consistently superior performance of assays targeting conserved non-structural proteins highlights a methodological strategy for reducing false negatives caused by genetic variation. Consequently, these insights are crucial for informing the development of more robust next-generation detection systems.

This systematic review and meta-analysis strengthens the evidence regarding the diagnostic accuracy of major PRRSV detection methods by synthesizing a substantial body of primary studies. Nevertheless, this study has certain limitations that should be considered. First, although it was not feasible to include all globally available studies due to objective constraints such as data accessibility and reporting variability, the comprehensive retrieval from multiple mainstream databases and the inclusion of publicly accessible literature have helped minimize this limitation. Second, while some included studies did not provide complete methodological details, such as specific detection targets or sample types, which restricted certain subgroup analyses, all available valid data were systematically extracted and analyzed to objectively reflect the current research landscape. Third, potential publication bias was suggested by funnel plot asymmetry; however, statistical evaluation using Egger’s test was performed to quantify and acknowledge this risk. Finally, although the geographic distribution of studies was skewed, with a predominance of data from China, this reflects regional research focus and was analytically accounted for in subgroup and meta-regression analyses. Future updates would benefit from more balanced global data, but the current findings remain valid within the represented contexts.

## Conclusion

Overall when developing new methods for detecting PRRSV, it is recommended to select non-structural proteins as detection targets. For laboratory testing of PRRSV, molecular amplification detection techniques are advised as the priority. In terms of sample collection, tissues—particularly lung tissues and lymph nodes—are recommended as the specimens to be tested. Additionally, this meta-analysis indicates that among the established PRRSV detection methods, emphasis should be placed on molecular amplification techniques, as they exhibit the highest performance in diagnosing PRRSV infections. Therefore, molecular amplification techniques are recommended as the preferred method for laboratory detection of PRRSV.

## Supplementary Information

Below is the link to the electronic supplementary material.

Supplementary Material 1

Supplementary Material 2

Supplementary Material 3

Supplementary Material 4

## Data Availability

Please contact author for data requests.

## Declarations

### Registration and protocol

This Meta-analysis has not yet been registered, and the study protocol can be obtained from the corresponding author upon reasonable request.

## Abbreviations

PRRSV: Porcine reproductive and respiratory syndrome virus
ELISA: Enzyme-linked immunosorbent assay
PCR: Polymerase chain reaction RT-PCR: reverse transcription-polymerase chain reaction
RT-qPCR: Reverse transcription-real-time fluorescent quantitative polymerase chain reaction TP: true positive
FP: False positive
TN: True negative
FN: False negative
