# Supplementary material for: Systematic review and meta-analysis on the diagnostic accuracy of various detection methods for porcine reproductive and respiratory syndrome virus
Source: Porcine Health Manag. 2026 Jan 14;12:4. doi: 10.1186/s40813-025-00482-1 (PMC12888294; doi:10.1186/s40813-025-00482-1)
Supplement: Supplementary file 3 — Supplementary Material 3 [file 40813_2025_482_MOESM3_ESM.docx]

Table S1. Description of studies included in the traditional immunoassay techniques

| Year of publication | Number of research samples | Sensitivity | Specificity | Detection target | Types of tissue samples | Comparative trial | First author | Country | References |
| --- | --- | --- | --- | --- | --- | --- | --- | --- | --- |
| 2015 | 170 | 1.000 | 0.926 | PRRSV Nsp2 | Serum samples | IDEXX | Y. H. Xiao | China | [1] |
| 2015 | 20 | 0.857 | 0.667 | PRRSV N | Serum samples | IDEXX | Qiwen Wang | China | [2] |
| 2015 | 859 | 0.962 | 0.959 | PRRSV N | Serum samples | IDEXX | Jin Zheng | China | [3] |
| 2015 | 37 | 0.889 | 0.929 | PRRSV N | Serum samples | IDEXX | Qiaona Niu | China | [4] |
| 2015 | 37 | 0.778 | 1.000 | PRRSV Nsp9 | Serum samples | IDEXX | Qiaona Niu | China | [4] |
| 2015 | 338 | 0.895 | 0.867 | PRRSV GP5 | Serum samples | IDEXX | Lisha Hao | China | [5] |
| 2015 | 497 | 0.941 | 0.886 | PRRSV N | Serum samples | IDEXX | Mingtan Sun | China | [6] |
| 2015 | 33 | 0.897 | 0.750 | PRRSV GP5 | Serum samples | IDEXX | Huan Yang | China | [7] |
| 2015 | 33 | 0.931 | 0.750 | PRRSV Nsp7 | Serum samples | IDEXX | Huan Yang | China | [7] |
| 2015 | 208 | 0.991 | 0.957 | PRRSV Nsp2 | Serum samples | IDEXX | Y. H. Xiao | China | [1] |
| 2016 | 80 | 0.938 | 0.667 | PRRSV N | Serum samples | IDEXX | Fuliang Cheng | China | [8] |
| 2016 | 288 | 0.931 | 0.909 | PRRSV Nsp10 | Serum samples | IDEXX | Jiaping Feng | China | [9] |
| 2016 | 505 | 0.938 | 0.760 | PRRSV GP5 | Serum samples | IDEXX | Y. Wang | China | [10] |
| 2017 | 150 | 0.881 | 0.907 | PRRSV Nsp7 | Serum samples | IDEXX | Hui Zhang | China | [11] |
| 2017 | 176 | 0.858 | 0.839 | PRRSV N | Serum samples | IDEXX | Ruosong Yang | China | [12] |
| 2017 | 208 | 0.951 | 0.905 | PRRSV Nsp2 | Serum samples | IDEXX | X.X. Wang | China | [13] |
| 2017 | 120 | 0.917 | 0.650 | PRRSV N | Serum samples | IDEXX | Jitka Frölichová | Czech Republic | [14] |
| 2017 | 120 | 0.724 | 0.886 | PRRSV M | Serum samples | IDEXX | Jitka Frölichová | Czech Republic | [14, 15] |
| 2017 | 1034 | 0.930 | 0.887 | PRRSV | Serum samples | IDEXX | Huawei Li | China | [15] |
| 2018 | 100 | 0.923 | 0.917 | PRRSV Nsp2 | Serum samples | IDEXX | Xulei Gao | China | [16] |
| 2018 | 36 | 0.833 | 0.958 | PRRSV N | Serum samples | IDEXX | Jun Wang | China | [17] |
| 2018 | 947 | 0.971 | 0.994 | PRRSV N | Serum samples | IDEXX | Ji Eun Yu | South Korea | [18] |
| 2018 | 247 | 0.984 | 1.000 | PRRSV N | Serum samples | IDEXX | Jie Li | China | [19] |
| 2018 | 247 | 0.936 | 1.000 | PRRSV Nsp1α | Serum samples | IDEXX | Jie Li | China | [19] |
| 2019 | 559 | 0.976 | 0.519 | PRRSV M | Serum samples | IDEXX | Wansheng Li | China | [20] |
| 2019 | 807 | 0.974 | 0.622 | PRRSV Nsp7 | Serum samples | IDEXX | Qun Li | China | [21] |
| 2019 | 60 | 0.889 | 0.867 | PRRSV Nsp7 | Serum samples | IDEXX | Junjie Yan | China | [22] |
| 2019 | 250 | 0.904 | 0.904 | PRRSV GP5 | Serum samples | IDEXX | Yuan Wang | China | [23] |
| 2019 | 196 | 0.647 | 0.963 | PRRSV N | Serum samples | IDEXX | Hansi Chen | China | [24] |
| 2019 | 125 | 0.933 | 0.950 | PRRSV Nsp2 | Serum samples | IDEXX | Xiaona Xiu | China | [25] |
| 2019 | 130 | 0.951 | 0.913 | PRRSV Nsp4 | Serum samples | IDEXX | Lei Liu | China | [26] |
| 2019 | 207 | 0.945 | 0.708 | PRRSV | Serum samples | IDEXX | Zezhou Wang | China | [27] |
| 2020 | 147 | 0.368 | 0.083 | PRRSV GP5 | Serum samples | IDEXX | Ziteng Deng | China | [28] |
| 2020 | 3325 | 0.934 | 0.973 | PRRSV NSP7/N | Serum samples | IDEXX | Yanqiu Wei | China | [29] |
| 2020 | 200 | 1.000 | 1.000 | PRRSV N | Serum samples | IDEXX | Yue Wu | China | [30] |
| 2021 | 240 | 0.809 | 0.908 | PRRSV Nsp12 | Serum samples | IDEXX | Xiaobo Zhang | China | [31] |
| 2021 | 303 | 0.871 | 0.908 | PRRSV GP5 | Serum samples | IDEXX | Wenping Xia | China | [32] |
| 2021 | 326 | 0.774 | 0.837 | PRRSV M | Serum samples | IDEXX | Jun Zhao | China | [33] |
| 2021 | 326 | 1.000 | 0.925 | PRRSV M | Serum samples | IDEXX | Jun Zhao | China | [33] |
| 2021 | 83 | 0.966 | 0.800 | PRRSV N | Serum samples | IDEXX | Liye Xia | China | [34] |
| 2022 | 211 | 0.914 | 0.759 | PRRSV | Serum samples | IDEXX | Jian Sun | China | [35] |
| 2022 | 117 | 0.960 | 0.611 | PRRSV Nsp7 | Serum samples | IDEXX | Mengmeng Zhao | China | [36] |
| 2022 | 217 | 1.000 | 0.984 | Cluster of differentiation 163 | Mixed samples | RT-PCR | Rajib Deb | India | [37] |
| 2022 | 108 | 0.739 | 0.903 | PRRSV | Mixed samples | RT-PCR | Wansheng Li | China | [38] |
| 2023 | 108 | 0.739 | 0.903 | PRRSV | Serum samples | IDEXX | Wansheng Li | China | [39] |
| 2023 | 333 | 0.984 | 0.905 | PRRSV N | Serum samples | IDEXX | Man Zhao | China | [40] |
| 2023 | 74 | 0.906 | 0.881 | PRRSV N | Serum samples | IDEXX | Peiyuan Gao | China | [41] |
| 2023 | 242 | 0.597 | 0.971 | PRRSV N | Mixed samples | PCR | Wansheng Li | China | [39] |
| 2023 | 184 | 0.914 | 0.909 | PRRSV N | Serum samples | IDEXX | Mingrui Liu | China | [42] |
| 2023 | 170 | 0.948 | 0.926 | PRRSV M | Serum samples | IDEXX | Ranran Hu | China | [43] |
| 2024 | 230 | 0.889 | 0.930 | PRRSV Nsp4 | Serum samples | IDEXX | Huiyang Sha | China | [44] |
| 2024 | 80 | 0.935 | 1.000 | PRRSV N | Serum samples | IDEXX | Ronglin Chen | China | [45] |
| 2024 | 1107 | 0.987 | 0.973 | PRRSV Nsp4 | Serum samples | IDEXX | Chaolun Fu | China | [46] |
| 2024 | 215 | 0.995 | 0.944 | PRRSV N | Serum samples | IDEXX | Chaolun Fu | China | [47] |
| 2024 | 189 | 0.967 | 0.979 | PRRSV N/M | Serum samples | IDEXX | Rui Yang | China | [48] |

The detection target in the chart is not specified by subtype and is assumed to be PRRSV-2 by default.

Table S2. Description of studies included in the molecular amplification detection techniques

| Year of publication | Number of research samples | Sensitivity | Specificity | Detection target | Types of tissue samples | Comparative trial | First author | Country | References |
| --- | --- | --- | --- | --- | --- | --- | --- | --- | --- |
| 2015 | 62 | 1.000 | 0.968 | PRRS | Tissue samples | RT-qPCR | Michael Eschbaumer | Canada | [49] |
| 2015 | 49 | 1.000 | 1.000 | PRRSV Nsp2 | Tissue samples | RT-qPCR | L. Hu | China | [50] |
| 2015 | 36 | 1.000 | 1.000 | PRRSV M | Tissue samples | RT-PCR | Hongguang Wang | China | [51] |
| 2015 | 175 | 1.000 | 1.000 | PRRSV 1/2 N | Tissue samples | RT-PCR | Xinyou Yu | China | [52] |
| 2015 | 61 | 1.000 | 1.000 | PRRSV Nsp1α | Mixed samples | PCR | Yuyan Chen | China | [53] |
| 2015 | 11 | 1.000 | 1.000 | PRRSV N | Tissue samples | PCR | Yi Zuo | China | [54] |
| 2015 | 49 | 1.000 | 1.000 | PRRSV Nsp2 | Tissue samples | PCR | Ling Hu | China | [55] |
| 2015 | 52 | 1.000 | 0.762 | PRRSV N | Tissue samples | RT-qPCR | Xiangfen Yuan | China | [56] |
| 2015 | 132 | 0.895 | 0.938 | PRRSV N | Tissue samples | PCR | Hiroshi ISEKI | Japan | [57] |
| 2016 | 85 | 0.959 | 0.972 | PRRSV 1 | Mixed samples | RT-qPCR | Ji-Young Park | South Korea | [58] |
| 2016 | 131 | 0.906 | 0.936 | PRRSV GP5 | Tissue samples | RT-PCR | Yimei Zhou | China | [59] |
| 2016 | 242 | 0.939 | 0.990 | PRRSV | Tissue samples | RT-PCR | Ru Chen | China | [60] |
| 2016 | 183 | 1.000 | 1.000 | PRRSV 2 Nsp2 | Serum samples | PCR | Bing Li | China | [61] |
| 2016 | 183 | 1.000 | 1.000 | PRRSV1 N | Serum samples | PCR | Bing Li | China | [61] |
| 2016 | 146 | 0.976 | 0.990 | PRRSV GP2 | Tissue samples | PCR | Ning Luo | China | [62] |
| 2016 | 39 | 1.000 | 1.000 | PRRSV N | Tissue samples | PCR | Lu Xiao | China | [63] |
| 2016 | 64 | 1.000 | 1.000 | PRRSV M | Tissue samples | PCR | Ling Hu | China | [64] |
| 2016 | 19 | 1.000 | 0.833 | PRRSV Nsp9 | Tissue samples | PCR | Mengmeng Zhao | China | [65] |
| 2016 | 63 | 0.963 | 0.972 | PRRSV 2 | Mixed samples | RT-qPCR | Ji-Young Park | South Korea | [58] |
| 2016 | 92 | 0.933 | 1.000 | H-PRRSV 2 | Mixed samples | RT-qPCR | Yang Yang | China | [66] |
| 2017 | 79 | 1.000 | 1.000 | PRRSV N | Tissue samples | RT -PCR | Xiaojing Li | China | [67] |
| 2017 | 125 | 0.916 | 1.000 | PRRSV | Serum samples | RT-PCR | Qi Yang | China | [68] |
| 2017 | 118 | 1.000 | 1.000 | PRRSV N | Mixed samples | PCR | Xiaowen Zheng | China | [69] |
| 2017 | 15 | 0.857 | 1.000 | PRRSV 2 | Tissue samples | PCR | Hye Kwon Kim | South Korea | [70] |
| 2017 | 62 | 1.000 | 0.800 | PRRSV 2 | Tissue samples | RT-PCR | Jian-chang Wang | China | [66] |
| 2018 | 183 | 0.838 | 0.904 | PRRSV | Tissue samples | RT-PCR | Xinyou Yu | China | [71] |
| 2018 | 68 | 0.960 | 1.000 | PRRSV N | Tissue samples | PCR | Feng Yang | China | [72] |
| 2018 | 18 | 1.000 | 1.000 | PRRSV N | Tissue samples | RT-PCR | Fengping Sun | China | [73] |
| 2018 | 120 | 0.868 | 1.000 | PRRSV N | Tissue samples | RT-PCR | Riteng Zhang | China | [74] |
| 2019 | 119 | 0.920 | 0.979 | PRRSV | Mixed samples | RT-qPCR | Yan Zhao | China | [75] |
| 2019 | 238 | 0.733 | 0.982 | PRRSV N | Mixed samples | RT-PCR | Xinyou Yu | China | [76] |
| 2019 | 73 | 1.000 | 1.000 | PRRSV GP2 | Mixed samples | RT-PCR | Yu Feng | China | [77] |
| 2019 | 117 | 1.000 | 1.000 | PRRSV | Mixed samples | RT-PCR | Yan Zhao | China | [75] |
| 2019 | 100 | 0.894 | 1.000 | PRRSV 1/2 | Tissue samples | RT-PCR | Ji-Young Park | South Korea | [78] |
| 2019 | 270 | 0.848 | 0.762 | PRRSV N | Mixed samples | PCR | Jie Zhang | China | [79] |
| 2019 | 96 | 1.000 | 1.000 | PRRSV N | Tissue samples | PCR | Shijie Lan | China | [80] |
| 2019 | 19 | 1.000 | 0.846 | PRRSV | Mixed samples | RT-PCR | Kai Wang | China | [81] |
| 2019 | 37 | 1.000 | 1.000 | PRRSV | Tissue samples | RT-qPCR | Yafei Chang | China | [82] |
| 2020 | 85 | 1.000 | 1.000 | PRRSV N | Tissue samples | PCR | Xiaona Xiu | China | [83] |
| 2020 | 33 | 1.000 | 0.750 | PRRSV N | Tissue samples | PCR | Lanlan Zheng | China | [84] |
| 2021 | 58 | 1.000 | 1.000 | PRRSV N | Mixed samples | RT-qPCR | Wataru Fukunaga | Japan | [85] |
| 2021 | 200 | 1.000 | 1.000 | PRRSV 2 GP5 | Serum samples | RT -PCR | Ya Gao | China | [86] |
| 2021 | 80 | 1.000 | 1.000 | PRRSV M | Tissue samples | RT -PCR | Yuhong Chen | China | [87] |
| 2021 | 1143 | 1.000 | 0.996 | PRRSV N | Tissue samples | RT-PCR | Yating Chen | China | [87] |
| 2021 | 846 | 0.953 | 0.989 | PRRSV 1/2 | Mixed samples | RT-PCR | Gaurav Rawal | United States | [88] |
| 2021 | 58 | 1.000 | 1.000 | PRRSV GP5 | Tissue samples | PCR | Xiaoxia Liao | China | [89] |
| 2021 | 54 | 0.833 | 0.979 | PRRSV M | Serum samples | PCR | Wang Zheng | China | [90] |
| 2021 | 11 | 1.000 | 1.000 | PRRSV Nsp2 | Tissue samples | RT-qPCR | Siyuan Liu | China | [91] |
| 2022 | 56 | 1.000 | 1.000 | PRRSV Nsp2 | Serum samples | RT-qPCR | Xinyou Yu | China | [92] |
| 2022 | 182 | 1.000 | 1.000 | PRRSV Nsp2 | Mixed samples | RT-qPCR | Lili Wang | China | [93] |
| 2022 | 47 | 0.882 | 0.933 | PRRSV Nsp1α、GP4 GP5 N | Tissue samples | RT-PCR | Fangzhou Wang | China | [94] |
| 2022 | 220 | 1.000 | 1.000 | PRRSV Nsp2 | Serum samples | RT-PCR | Liuyang Zhao | China | [95] |
| 2022 | 220 | 1.000 | 1.000 | PRRSV Nsp2 | Serum samples | RT-PCR | Liuyang Zhao | China | [95] |
| 2022 | 67 | 1.000 | 1.000 | PRRSV M/N | Tissue samples | RT-PCR | Mengjie Wang | China | [96] |
| 2022 | 312 | 0.890 | 0.950 | PRRSV N | Tissue samples | RT-PCR | Kaichuang Shi | China | [97] |
| 2022 | 30 | 1.000 | 1.000 | PRRSV N | Tissue samples | PCR | Yuanhang Zhang | China | [98] |
| 2022 | 144 | 1.000 | 1.000 | PRRSV M | Tissue samples | PCR | Bin Liu | China | [99] |
| 2022 | 87 | 1.000 | 1.000 | PRRSV M | Mixed samples | RT-qPCR | Wenlong Xia | China | [100] |
| 2022 | 87 | 0.957 | 1.000 | PRRSV M | Mixed samples | RT-qPCR | Wenlong Xia | China | [100] |
| 2023 | 62 | 1.000 | 1.000 | PRRSV N | Tissue samples | RT-qPCR | Xinyou Yu | China | [101] |
| 2023 | 300 | 1.000 | 0.992 | PRRSV GP2 | Tissue samples | RT-qPCR | Xin Li | China | [102] |
| 2023 | 511 | 0.884 | 0.992 | PRRSV 1 Nsp1α | Serum samples | RT-qPCR | Bangjun Gong | China | [103] |
| 2023 | 509 | 1.000 | 0.994 | PRRSV 2 Nsp1α | Serum samples | RT-qPCR | Bangjun Gong | China | [103] |
| 2023 | 100 | 0.960 | 0.973 | PRRSV 1/2 | Mixed samples | RT-PCR | Shengnan Ruan | China | [104] |
| 2023 | 576 | 1.000 | 1.000 | PRRSV GP5 | Mixed samples | RT-PCR | Xuezhen Cao | China | [105] |
| 2023 | 53 | 0.923 | 0.975 | PRRSV Nsp2 | Serum samples | RT-PCR | Lei Chen | China | [106] |
| 2023 | 64 | 1.000 | 1.000 | PRRSV N | Tissue samples | RT-PCR | Yingping Wang | China | [107] |
| 2023 | 45 | 1.000 | 1.000 | PRRSV N | Mixed samples | PCR | Yuxin Zhang | China | [108] |
| 2023 | 85 | 1.000 | 1.000 | PRRSV N | Tissue samples | PCR | Xukun Men | China | [109] |
| 2023 | 34 | 1.000 | 1.000 | PRRSV M | Tissue samples | PCR | Bin Li | China | [110] |
| 2023 | 168 | 1.000 | 1.000 | PRRSV N | Tissue samples | PCR | Qian Zhou | China | [111] |
| 2023 | 168 | 1.000 | 1.000 | PRRSV Nsp2 | Tissue samples | PCR | Qian Zhou | China | [111] |
| 2023 | 92 | 1.000 | 1.000 | PRRSV M | Tissue samples | PCR | Shuo Zhao | China | [112] |
| 2023 | 39 | 1.000 | 1.000 | PRRSV M | Mixed samples | RT-PCR | Yao Chen | China | [113] |
| 2023 | 160 | 1.000 | 0.979 | PRRSV GP2a | Serum samples | PCR | Suzhen Chen | China | [114] |
| 2023 | 520 | 1.000 | 0.905 | PRRSV N/Nsp2 | Serum samples | RT-qPCR | Qian Zhou | China | [115] |
| 2023 | 148 | 0.950 | 1.000 | PRRSV | Tissue samples | RT-qPCR | Chihai Ji | China | [116] |
| 2023 | 50 | 1.000 | 1.000 | PRRSV | Serum samples | RT-qPCR | Diem Hong Tran | Vietnam | [117] |
| 2024 | 298 | 1.000 | 0.992 | PRRSV GP2 | Tissue samples | RT-qPCR | Xin Li | China | [118] |
| 2024 | 88 | 1.000 | 1.000 | PRRSV N | Serum samples | RT-qPCR | Tingting Sun | China | [119] |
| 2024 | 3129 | 0.991 | 0.995 | PRRSV M | Tissue samples | RT-qPCR | Zhuo Feng | China | [120] |
| 2024 | 4932 | 0.990 | 0.995 | PRRSV N | Tissue samples | RT-qPCR | Yan Ma | China | [121] |
| 2024 | 104 | 0.974 | 0.963 | PRRSV M/Nsp2 | Mixed samples | RT-qPCR | Chunhao Tao | China | [122] |
| 2024 | 30 | 1.000 | 1.000 | PRRSV M | Mixed samples | RT-qPCR | Geng Wang | China | [123] |
| 2024 | 155 | 1.000 | 1.000 | PRRSV N、M | Tissue samples | RT-PCR | Yi Xu | China | [124] |
| 2024 | 130 | 1.000 | 1.000 | PRRSV NSP2 | Tissue samples | RT-PCR | Ying Liu | China | [125] |
| 2024 | 30 | 1.000 | 1.000 | PRRSV GP5 | Tissue samples | PCR | Yang Cheng | China | [126] |
| 2024 | 62 | 1.000 | 0.911 | PRRSV N | Tissue samples | PCR | Lele An | China | [127] |
| 2024 | 161 | 0.940 | 1.000 | PRRSV N | Serum samples | PCR | Na Yu | China | [128] |
| 2024 | 808 | 0.971 | 0.991 | PRRSV Nsp7 | Serum samples | PCR | Na Yu | China | [129] |

The detection target in the chart is not specified by subtype and is assumed to be PRRSV-2 by default.

Table S3. Description of studies included in the convergent diagnostic technologies

| Year of publication | Number of research samples | Sensitivity | Specificity | Detection target | Types of tissue samples | Comparative trial | First author | Country | References |
| --- | --- | --- | --- | --- | --- | --- | --- | --- | --- |
| 2015 | 189 | 0.892 | 0.968 | PRRSV | Mixed samples | RT-qPCR | Ru Chen | China | [130] |
| 2015 | 47 | 0.857 | 0.939 | PRRSV M | Tissue samples | RT-PCR | Hongchao Gou | China | [131] |
| 2019 | 16 | 1.000 | 1.000 | PRRSV N | Tissue samples | PCR | Shengli Liu | China | [132] |
| 2019 | 100 | 1.000 | 1.000 | PRRSV 1/2 | Tissue samples | PCR | Ji-Young Park | Korea | [78] |
| 2019 | 148 | 0.978 | 1.000 | PRRSV | Tissue samples | RT-qPCR | Xulong Wu | China | [133] |
| 2019 | 16 | 1.000 | 1.000 | PRRSV | Tissue samples | PCR | Shengli Liu | China | [134] |
| 2022 | 50 | 1.000 | 1.000 | PRRSV Nsp2 | Serum samples | RT-PCR | Yueyan Zeng | China | [135] |
| 2022 | 108 | 0.739 | 0.903 | PRRSV N | Mixed samples | PCR | Wansheng Li | China | [136] |
| 2023 | 80 | 1.000 | 1.000 | PRRSV N | Serum samples | RT-qPCR | Junyu Bi | China | [137] |
| 2023 | 160 | 1.000 | 0.968 | PRRSV GP2a | Serum samples | PCR | Suzhen Chen | China | [138] |
| 2023 | 32 | 0.900 | 0.833 | PRRSV | Serum samples | PCR | Hui Zheng | China | [139] |
| 2023 | 65 | 1.000 | 1.000 | PRRSV N | Tissue samples | PCR | Xulong Wu | China | [140] |
| 2024 | 121 | 0.938 | 1.000 | PRRSV 3'-UTR | Tissue samples | RT-qPCR | JingxueYu | China | [141] |
| 2024 | 157 | 1.000 | 0.978 | PRRSV N | Serum samples | PCR | Ziwei Zhang | China | [142] |

The detection target in the chart is not specified by subtype and is assumed to be PRRSV-2 by default.

References

1. Xiao YH, Wang TT, Zhao Q, Wang CB, Lv JH, Nie L, Gao JM, Ma XC, Hsu WH, Zhou EM: **Development of indirect ELISAs for differential serodiagnosis of classical and highly pathogenic porcine reproductive and respiratory syndrome virus**. *Transbound Emerg Dis* 2014, **61**(4):341-349.

2. Wang QW, Zhang HX, Gao ZQ, Lin XC, Zhang LC. **Prokaryotic expression of porcine reproductive and respiratory syndrome virus N protein and establishment of an indirect ELISA method**. China, *Chinese Journal of Veterinary Medicine*. 2015;51(11):49-52.

3. Zheng J. **Comparative study on three detection methods for PRRSV antibodies and establishment of an IgM antibody detection method**. China, Master's thesis. 2015.

4. Niu QN. **Establishment and application of two indirect ELISA methods for the detection of PRRSV antibodies.** China, Master's thesis. 2015.

5. Hao LS. **High-efficiency secretory expression of HP-PRRSV GP5 protein in yeast and establishment of an iELISA method for antibody detection**. China, Master's thesis. 2015.

6. Sun MT. **Prokaryotic expression of ORF7 gene of Hebei local strain of porcine reproductive and respiratory syndrome virus and establishment of an ELISA method**. China, Master's thesis. 2015.

7. Yang H. **Prokaryotic expression of GP5 and Nsp7 of PRRSV and establishment of an ELISA detection method**. China, Master's thesis. 2015.

8. Cheng FL, Li FH, Liu YQ, Nie ZJ, Chen TT, Fang D, Fan MN, Gu W, Wang CF. **Establishment and application of an indirect ELISA detection method for porcine reproductive and respiratory syndrome virus recombinant N protein**. China, *China Animal Husbandry & Veterinary Medicine*, 2016, 43(11):2900-2906.

9. Feng JP. **Prokaryotic expression of PRRSV non-structural protein Nsp10 and preliminary establishment of an indirect ELISA detection method**. China, Master's thesis. 2016.

10. Wang Y, Guo J, Qiao S, Li Q, Yang J, Jin Q, Zhang G: **GP5 Protein-based ELISA for the Detection of PRRSV Antibodies**. *Pol J Vet Sci* 2016, **19**(3):495-501.

11. Zhang H, Yang H, Chang XB, Yin J, Song M, Qi L, Cui HZ. **Establishment of an NSP7-ELISA detection method for porcine reproductive and respiratory syndrome virus antibodies**. China, *Heilongjiang Animal Science and Veterinary Medicine*, 2017(01):174-176.

12. Yang RS, Qi XM, Xiong YT. Establishment of an indirect ELISA for detection of porcine reproductive and respiratory syndrome virus. China, *Food Safety Guide*. 2017(19):64-67.

13. Wang XX, Wang FX, Li ZG, Wen YJ, Wang X, Song N, Wu H: **Development of an indirect enzyme-linked immunosorbent assay (ELISA) to differentiate antibodies against wild-type porcine reproductive and respiratory syndrome from the vaccine strain TJM-F92 based on a recombinant Nsp2 protein**. *J Virol Methods* 2018, **251**:151-154.

14. Frolichova J, Molinkova D, Sedlinska M, Celer V: **Expression and diagnostic use of recombinant M protein of the porcine reproductive and respiratory syndrome virus**. *ACTA VETERINARIA BRNO* 2017, **86**(1):11-17.

15. Li H, Yang J, Bao D, Hou J, Zhi Y, Yang Y, Ji P, Zhou E, Qiao S, Zhang G: **Development of an immunochromatographic strip for detection of antibodies against porcine reproductive and respiratory syndrome virus**. *J Vet Sci* 2017, **18**(3):307-316.

16. Gao XL, Sun GQ, Li YF. **Establishment of an indirect ELISA for detection of antibodies against highly pathogenic porcine reproductive and respiratory syndrome virus.** China, *China Animal Health Inspection*. 2018, 35(11):75-78.

17. Wang J, Dai JF, Ma B, Ding YZ, Ou YW, Liu YS, Zhao LH, Zhang YG, Zhang J. **Prokaryotic soluble expression of porcine reproductive and respiratory syndrome virus nucleocapsid protein and preliminary establishment of an indirect ELISA for antibody detection**. China, *Chinese Veterinary Science*. 2018, **48**(04):419-427.

18. Yu JE, Ouh IO, Kang H, Lee HY, Cheong KM, Cho IS, Cha SH: **An enhanced immunochromatographic strip test using colloidal gold nanoparticle-labeled dual-type N proteins for detection of antibodies to PRRS virus**. *J Vet Sci* 2018, **19**(4):519-527.

19. Li J, Wang G, Yang D, Zhao B, Zhao Y, Liu Y, Cai X, Nan Y, Zhou E-M, Wu C: **Development of luciferase-linked antibody capture assay based on luciferase immunoprecipitation systems for antibody detection of porcine reproductive and respiratory syndrome virus**. *BMC BIOTECHNOLOGY* 2018, **18**.

20. Li WS, Li MH, Yang JW, Tian ZJ, Wang Q, Leng CL. **Establishment and preliminary application of a competitive ELISA for detection of antibodies against North American genotype porcine reproductive and respiratory syndrome virus**. China, *Chinese Journal of Preventive Veterinary Medicine*. 2019, 41(12):1221-1226.

21. Li Q, Fan J, Wei RR, Ye ZQ, Ding GW. **Establishment of an indirect ELISA for detection of antibodies against porcine reproductive and respiratory syndrome virus.** China, *Foreign Animal Husbandry (Pigs and Poultry)*. 2019, 39(08):12-18.

22. Yan JJ. **Preparation of monoclonal antibodies against porcine reproductive and respiratory syndrome virus and establishment of an indirect ELISA method.** China, *Master's thesis*. 2019.

23. Wang Y. **Prokaryotic expression of PRRSV GP5 protein and establishment of an indirect ELISA antibody detection method**. China, *Master's thesis*. 2019.

24. Chen HS. **Establishment of an indirect ELISA method for PRRSV N protein**. China, *Master's thesis*. 2019.

25. Xiu XN, Zhang SL, Shen ZQ, Liu L, Ma YB. **Establishment of an ELISA for PRRSV antibody detection using recombinant Nsp2-399 protein as coating antige**n. China, *Heilongjiang Animal Science and Veterinary Medicine*. 2019(16):76-80.

26. Liu L, Ma YB, Wang WX, Li F, Shen ZQ, Zhang SL. **Establishment of an ELISA for detection of antibodies against PRRSV Nsp4 protein**. China, *Progress in Veterinary Medicine*. 2019, 40(07):9-13.

27. Wang ZZ, Zhang Y, Wu JQ, Zhang J, Chen DS, He DM. **Development of a highly sensitive fluorescent immunochromatographic rapid test kit for PRRS antibody detection**. China, *Sichuan Animal & Veterinary Sciences*. 2019, 46(11):27-29+32.

28. Deng ZT. **Establishment of an indirect ELISA for PRRSV GP5 protein antibody detection and development of monoclonal antibodies**. China, *Master's thesis*. 2020.

29. Wei Y, Yang B, Li Y, Duan Y, Tian D, He B, Chen C, Liu W, Yang L: **A rapid and quantitative fluorescent microsphere immunochromatographic strip test for detection of antibodies to porcine reproductive and respiratory syndrome virus**. *J Vet Sci* 2020, **21**(4):e68.

30. Wu Y, Wu X, Chen J, Hu J, Huang X, Zhou B: **A novel protein chip for simultaneous detection of antibodies against four epidemic swine viruses in China**. *BMC Vet Res* 2020, **16**(1):162.

31. Zhang XB, Feng P, Zhao Q, Zhou EM. **Prokaryotic expression of porcine reproductive and respiratory syndrome virus Nsp12 and establishment of an indirect ELISA method**. China, *Progress in Veterinary Medicine*. 2021, 42(11):48-52.

32. Xia WP. **Preparation of PRRSV GP5 recombinant protein and establishment and application of an indirect ELISA antibody detection method**. China, *Master's thesis*. 2021.

33. Zhao J, Zhang R, Zhu L, Deng H, Li F, Xu L, Huan J, Sun X, Xu Z: **Establishment of a peptide-based enzyme-linked immunosorbent assay for detecting antibodies against PRRSV M protein**. *BMC Vet Res* 2021, **17**(1):355.

34. Xia LY, Jiang Y, Shan H, Li GM. **Establishment of a liquid chip detection method for antibodies against porcine reproductive and respiratory syndrome virus**. China, *Journal of Agricultural Biotechnology*. 2021, 29(01):188-197.

35. Sun J. **Establishment and preliminary application of an ELISA method for detecting PRRSV antibodies using synthetic peptides as antigens**. China, *Chinese Journal of Animal Infectious Diseases*. 2022, 30(04):190-196.

36. Zhao MM, Sha HY, Zhang H, Huang LZ. **Establishment of an indirect ELISA for detection of PRRSV-2 NSP7**. China, *Journal of Foshan University (Natural Science Edition)*. 2022, 40(03):51-56.

37. Deb R, Yadav AK, Sengar GS, Sonowal J, Lalita D, Pegu SR, Singh I, Linda N, Das PJ, Kumar S *et al*: **Development of CD163 receptor-based enzyme-linked immunosorbent assay for diagnosis of porcine reproductive and respiratory syndrome virus**. *3 Biotech* 2022, **12**(11):325.

38. Li W, Li M, Zhang H, Li C, Xu H, Gong B, Fu J, Guo Z, Peng J, Zhou G *et al*: **A Novel Immunochromatographic Strip Based on Latex Microspheres for the Rapid Detection of North American-Type Porcine Reproductive and Respiratory Syndrome Virus**. *Front Microbiol* 2022, **13**:882112.

39. Li WS. **Establishment and application of PRRSV-2 antigen and antibody detection methods based on monoclonal antibodies**. China, *PhD dissertation*. 2023.

40. Zhao M. **Establishment and preliminary application of a competitive ELISA for PRRSV antibody detection**. China, *Master's thesis*. 2023.

41. Gao PY. **Establishment and application of an indirect ELISA method for porcine reproductive and respiratory syndrome virus N protein**. China, *Master's thesis*. 2023.

42. Liu MR, Yin XD, Yang XF, Lyu YY. **Establishment of a fluorescent microsphere-based antibody detection method for porcine reproductive and respiratory syndrome virus**. China, *China Animal Health Inspection*. 2023, 40(10):100-105.

43. Hu RR, Zhao YH, Liu Y, Zhang S, Guo Y, Zuo YZ, Fan JH. **Truncated expression of PRRSV M gene and establishment of a colloidal gold immunochromatographic assay**. China, *Chinese Journal of Animal Infectious Diseases*. 2023, 31(06):94-100.

44. Sha HY. **Genetic evolution analysis of PRRSV NSP4 and establishment of an indirect ELISA detection method**. China, *Master's thesis*. 2024..

45. Chen RL, Yan JC, Zhu SQ, Wang SY, Wang J, Gao SL, Xing YR, Liu QY, Zhu YX, Li JN, et al. **Prokaryotic expression of porcine reproductive and respiratory syndrome virus N protein and establishment of an indirect ELISA method**. China, *Chinese Journal of Animal Infectious Diseases*:1-12.

46. Fu C, Shao Q, Zhang L, Cui X, Chen T, Tian C, Qian F, Chu X, Li Y, Yang P *et al*: **Development of an enzyme-linked immunosorbent assay using a monoclonal antibody to a dominant epitope in non-structural protein 4 of porcine reproductive and respiratory syndrome virus**. *J Immunol Methods* 2024, **530**:113697.

47. Li W, Li Y, Li M, Zhang H, Feng Z, Xu H, Li C, Guo Z, Gong B, Peng J *et al*: **Development and application of a blocking ELISA based on a N protein monoclonal antibody for the antibody detection against porcine reproductive and respiratory syndrome virus 2**. *Int J Biol Macromol* 2024, **269**(Pt 2):131842.

48. Yang R, Ru Y, Wang H, Hao R, Li Y, Zhang T, Zheng H, Zhang Y, Zhao X: **Quantum dot fluorescent microsphere-based immunochromatographic strip for detecting PRRSV antibodies**. *Appl Microbiol Biotechnol* 2024, **108**(1):283.

49. Eschbaumer M, Li W, Wernike K, Marshall F, Czub M: **Probe-free real-time reverse transcription polymerase chain reaction assays for the detection and typing of porcine reproductive and respiratory syndrome virus in Canada**. *CANADIAN JOURNAL OF VETERINARY RESEARCH-REVUE CANADIENNE DE RECHERCHE VETERINAIRE* 2015, **79**(3):170-179.

50. Hu L, Lin XY, Yang ZX, Yao XP, Li GL, Peng SZ, Wang Y: **A multiplex PCR for simultaneous detection of classical swine fever virus, African swine fever virus, highly pathogenic porcine reproductive and respiratory syndrome virus, porcine reproductive and respiratory syndrome virus and pseudorabies in swines**. *Pol J Vet Sci* 2015, **18**(4):715-723.

51. Wang HG. **Establishment and application of multiplex PCR method for porcine respiratory viral diseases**. China, *Master's thesis*. 2015.

52. Yu XY, Li TZ, Wang JL, Tang N, Li F, Shen ZQ. **Establishment and application of a one-step duplex RT-PCR for rapid detection of American and European genotypes of PRRSV**. China, *Swine Industry Science*. 2015, 32(04):136-138.

53. Chen YY, Yang J, Nie FP, Wang Y, Zhang JL, Qin M, Xiao JW, Wang GM, Li YG, Cai JL. **Establishment and application of a multiplex PCR for detection of four pathogens associated with porcine respiratory disease complex**. China, *Chinese Veterinary Science*. 2015, 45(04):339-344.

54. Zuo Y, Wang JY, Yuan WZ, Sun JG. **Establishment of a multiplex PCR for detection of PCV2, PRV, PRRSV and CSFV**. China, *Chinese Veterinary Science*. 2015, 45(08):771-775.

55. Hu L, Wang Y, Yang ZX, Yao XP, Lin XY, Li GL. **Development of a multiplex PCR for simultaneous detection of five viruses causing reproductive failure in swine**. China, *Chinese Veterinary Science*. 2015, 45(10):1047-1052.

56. Yuan XF, Wu SQ, Lyu JZ, Zhang YN, Lin XM. **Establishment and application of a duplex RT-LAMP for detection of classical swine fever virus and porcine reproductive and respiratory syndrome virus**. China, *Chinese Veterinary Science*. 2015, 45(07):721-728.

57. Iseki H, Takagi M, Kuroda Y, Katsuda K, Mikami O, Tsunemitsu H, Yamakawa M: **Application of a SYBR®Green one step real-time RT-PCR assay to detect type 1 porcine reproductive and respiratory syndrome virus**. *J Vet Med Sci* 2014, **76**(10):1411-1413.

58. Park JY, Park S, Park YR, Kang DY, Kim EM, Jeon HS, Kim JJ, Kim WI, Lee KT, Kim SH *et al*: **Reverse-transcription loop-mediated isothermal amplification (RT-LAMP) assay for the visual detection of European and North American porcine reproductive and respiratory syndrome viruses**. *J Virol Methods* 2016, **237**:10-13.

59. Zhou YM, Lao XJ, Huang QL, Xu LN, Zheng Y, Ye X, Wang XD, Shao CY, Yang YC, Song HH. **Establishment and preliminary application of a fluorescent quantitative RT-PCR method for PRRSV detection**. *Animal Husbandry & Veterinary Medicine*. 2016, 48(01):34-39.

60. Chen R, Gao XB, Yu XL, Song CX, Qiu Y: **Novel multiplex PCR assay using locked nucleic acid (LNA)-based universal primers for the simultaneous detection of five swine viruses**. *J Virol Methods* 2016, **228**:60-66.

61. Li B, Liu LY, Lu H, Han J, Jiang H, Zhou ZT, Gao SY, Zhou TZ. **Establishment of a multiplex PCR detection method for three strains of porcine reproductive and respiratory syndrome virus**. China, *Chinese Journal of Veterinary Science*. 2016, 36(03):373-377+383.

62. Luo N, Wang DD, Yang ZT, Sun J, Xu SZ, Wang SC, Yin YB, Xu B. **Establishment and application of a multiplex PCR method for detection of six common swine viruses**. China, *Progress in Veterinary Medicine*. 2016, 37(12):1-6.

63. Xiao L, Wu XL, Wang Y, Yang ZX, Yao XP, Ren MS, Zhang PF, Leng YY, Zhang B, Liu YD, et al. **Development and preliminary application of a simplified target-enriched multiplex PCR for simultaneous detection of six swine viral diseases**. China, *Chinese Veterinary Science*. 2016, 46(06):695-701.

64. Hu L. **Establishment of 5-plex PCR and 7-plex GeXP-PCR detection methods for pathogens causing porcine reproductive disorders**. China, *Master's thesis*. 2016.

65. Zhao MM, Feng SL, Wang WJ, Xing X, Feng JP, Zhang GH. **Establishment of a real-time fluorescent quantitative PCR for Nsp9 gene and its expression dynamics in infected cells**. China, *China Animal Husbandry & Veterinary Medicine*. 2016, 43(10):2534-2540.

66. Yang Y, Qin X, Sun Y, Chen T, Zhang Z: **Rapid detection of highly pathogenic porcine reproductive and respiratory syndrome virus by a fluorescent probe-based isothermal recombinase polymerase amplification assay**. *Virus Genes* 2016, **52**(6):883-886.

67. Li XJ, Gong SY, Chen YQ, Li YM, Cai Y, Xu YF, Zhu L, Xu ZW. **Establishment and application of a duplex RT-PCR for detection of porcine reproductive and respiratory syndrome virus and porcine epidemic diarrhea virus**. China, *Chinese Veterinary Science*. 2017, 47(05):544-550.

68. Yang Q, Xi J, Chen X, Hu S, Chen N, Qiao S, Wan S, Bao D: **The development of a sensitive droplet digital PCR for quantitative detection of porcine reproductive and respiratory syndrome virus**. *Int J Biol Macromol* 2017, **104**(Pt A):1223-1228.

69. Zheng XW, Rao PB, Cai Y, Yang ZQ, Jiang YH. **Establishment and application of an EvaGreen real-time PCR method for detection of porcine reproductive and respiratory syndrome virus (PRRSV)**. China, *Jiangsu Agricultural Sciences*. 2017, 45(09):123-126.

70. Kim HK, Lyoo KS, Huynh TML, Moon HJ, Nguyen VG, Park BK: **Duplex nested reverse transcriptase polymerase chain reaction for simultaneous detection of type 2 porcine reproductive and respiratory syndrome virus and porcine circovirus type 2 from tissue samples**. *J Vet Sci* 2017, **18**(2):253-256.

71. Yu XY, Li TZ. **Establishment and application of a one-step duplex fluorescent RT-PCR for detection of classical swine fever virus and porcine reproductive and respiratory syndrome virus**. China, *China Animal Health Inspection*. 2018, 35(04):88-91.

72. Yang F. **Establishment and preliminary application of TaqMan multiplex fluorescent quantitative PCR for detection of four common swine viruses and four bacteria**. China, *Master's thesis*. 2018.

73. Sun FP, Gao J, Liu CQ, Li H, Yi JZ, Yao HJ. **Establishment and preliminary application of an RT-LAMP assay for porcine reproductive and respiratory syndrome virus**. China, *Shanghai Journal of Animal Husbandry and Veterinary Medicine*. 2018(01):2-5.

74. Zhang RT, Zhang QY, Jiang CL, Liu ZX, Yang MC, Jiang P, Bai J. **Establishment and application of a visual RT-LAMP assay for porcine reproductive and respiratory syndrome virus**. China, *Chinese Veterinary Science*. 2018, 48(01):7-12.

75. Zhao Y, Liu F, Li Q, Wu M, Lei L, Pan Z: **A multiplex RT-PCR assay for rapid and simultaneous detection of four RNA viruses in swine**. *J Virol Methods* 2019, **269**:38-42.

76. Yu XY, Li TZ, Wang YM, Xiu XN. **Establishment of a duplex TaqMan real-time fluorescent quantitative RT-PCR for detection of porcine reproductive and respiratory syndrome virus and porcine epidemic diarrhea virus**. China, *Heilongjiang Animal Science and Veterinary Medicine*. 2019(19):86-89+92.

77. Feng Y, Yang XY, Zhao J, Li YM, Xu ZW, Zhu L. **Establishment and application of a duplex RT-PCR for detection of porcine reproductive and respiratory syndrome virus and atypical porcine pestivirus**. China, *Chinese Journal of Preventive Veterinary Medicine*. 2019, 41(02):156-160.

78. Park JY, Kim SH, Lee KK, Kim YH, Moon BY, So B, Park CK: **Differential detection of porcine reproductive and respiratory syndrome virus genotypes by a fluorescence melting curve analysis using peptide nucleic acid probe-mediated one-step real-time RT-PCR**. *J Virol Methods* 2019, **267**:29-34.

79. Li F, Zeng YB, Jiang CY, Zhang RB, Peng KN, Chen B, Zhu L, Xu ZW, Cheng SC. **Establishment and application of a SYBR Green Ⅰ-based fluorescent quantitative RT-PCR for detection of porcine epidemic diarrhea virus**. China, *Chinese Veterinary Science*. 2021, 51(11):1355-1360.

80. Lan SJ, Chen L, Miao Y, Feng WY, Wang ZQ, Zhu QH, Jin ZH, Li Y, Xu X, Li D. **Establishment and preliminary application of a multiplex PCR for detection of CSFV, PRRSV and PCV2**. China, *China Swine Industry*. 2019, 14(06):22-25+30.

81. Wang K, Yang F, Luo LH, Wang WH, Li XX, Zhou HC, Fan ZX, Tian HL, Feng QW, Guo KK. **Establishment of a visual RT-LAMP method for detection of highly pathogenic and classical strains of porcine reproductive and respiratory syndrome virus**. China, *Chinese Journal of Veterinary Science*. 2019, 39(09):1660-1666+1673.

82. Chang Y, Deng Y, Li T, Wang J, Wang T, Tan F, Li X, Tian K: **Visual detection of porcine reproductive and respiratory syndrome virus using CRISPR-Cas13a**. *Transbound Emerg Dis* 2020, **67**(2):564-571.

83. Xiu XN, Li TZ, Yu XY. **Establishment of a duplex PCR for detection of porcine reproductive and respiratory syndrome virus and Mycoplasma hyorhinis**. China, *Chinese Journal of Veterinary Medicine*. 2020, 56(05):16-19+24.

84. Zheng LL, Chai LY, Tian RB, Zhao Y, Chen HY, Wang ZY: **Simultaneous detection of porcine reproductive and respiratory syndrome virus and porcine circovirus 3 by SYBR Green І-based duplex real-time PCR**. *Mol Cell Probes* 2020, **49**:101474.

85. Fukunaga W, Hayakawa-Sugaya Y, Koike F, Van Diep N, Kojima I, Yoshida Y, Suda Y, Masatani T, Ozawa M: **Newly-designed primer pairs for the detection of type 2 porcine reproductive and respiratory syndrome virus genes**. *J Virol Methods* 2021, **291**:114071.

86. Gao Y, Wu XX, Jiang XL, He XS, Xiao X, Zhou SH. **Establishment of a duplex RT-PCR for detection of North American and European genotypes of PRRSV**. China, *Journal of Beijing University of Agriculture*. 2021, 36(04):76-80.

87. Chen Y, Shi K, Liu H, Yin Y, Zhao J, Long F, Lu W, Si H: **Development of a multiplex qRT-PCR assay for detection of African swine fever virus, classical swine fever virus and porcine reproductive and respiratory syndrome virus**. *J Vet Sci* 2021, **22**(6):e87.

88. Rawal G, Yim-Im W, Chamba F, Smith C, Okones J, Francisco C, Zhang J: **Development and validation of a reverse transcription real-time PCR assay for specific detection of PRRSGard vaccine-like virus**. *Transbound Emerg Dis* 2022, **69**(3):1212-1226.

89. Liao XX, Tian ZG, Hu XL, Li YC. **Establishment and application of a duplex PCR for detection of porcine reproductive and respiratory syndrome virus and pseudorabies virus**. China, *Animal Husbandry and Veterinary Science (Electronic Edition)*. 2021(03):1-4.

90. Wang Z, Xie CZ, Li TY, Li ZX, Yu T, Yu CD, Zhang XM, Lu HJ, Jin NY. **Establishment and application of a fluorescent quantitative PCR assay for detection of NADC30-like lineage porcine reproductive and respiratory syndrome virus**. China, *Chinese Journal of Preventive Veterinary Medicine*. 2021, 43(05):495-500.

91. Liu S, Tao D, Liao Y, Yang Y, Sun S, Zhao Y, Yang P, Tang Y, Chen B, Liu Y *et al*: **Highly Sensitive CRISPR/Cas12a-Based Fluorescence Detection of Porcine Reproductive and Respiratory Syndrome Virus**. *ACS Synth Biol* 2021, **10**(10):2499-2507.

92. Yu XY, Li TZ, Xiao YQ, Shen ZQ. **Establishment of a nucleic acid extraction-free fluorescent quantitative RT-PCR for detection of PRRSV NADC30-like strains**. China, *Heilongjiang Animal Science and Veterinary Medicine*. 2022(14):74-77+83.

93. Wang LL, Lu C, Li FQ, Yan MH, Ren WK, Jiang S, Chi JJ, Zhang L, Li C, Li XL. **Establishment and application of a quadruple fluorescent quantitative PCR for detection of CSFV, PRRSV, PEDV and PRV**. China, *Chinese Veterinary Science*. 2022, 52(10):1259-1267.

94. Wang FZ, Zhang J, Ma XQ, Li PH, Bao HF, Wang J, Zhao ZX, Li JY, Li GX, Liu ZX, et al. **Establishment and application of a one-step multiplex RT-PCR for detection of porcine reproductive and respiratory syndrome virus**. China, *Chinese Veterinary Science*. 2022, 52(07):805-814.

95. Zhao LY, Li HQ, Rui X, Duan YF, He XS, Zhou SH. **Establishment of a duplex RT-PCR for detection of classical and highly pathogenic strains of porcine reproductive and respiratory syndrome virus**. China, *Journal of Beijing University of Agriculture*. 2022, 37(01):61-65.

96. Wang MJ, Zhang WL, Wang XR, Chen JX, Xia CY, Chen HY, Zhang H, Wang YE. **Establishment and preliminary application of a triplex RT-PCR for detection of porcine reproductive and respiratory syndrome virus, porcine circovirus type 3, and swine influenza virus**. China, *Microbiology China*. 2022, 49(12):5092-5099.

97. Shi K, Chen Y, Yin Y, Long F, Feng S, Liu H, Qu S, Si H: **A Multiplex Crystal Digital PCR for Detection of African Swine Fever Virus, Classical Swine Fever Virus, and Porcine Reproductive and Respiratory Syndrome Virus**. *Front Vet Sci* 2022, **9**:926881.

98. Zhang YH, Dong XY, Zhang YQ, Liu XC, Li HX, Chen XM, Pan JJ, Chen HY. **Establishment of a duplex SYBR Green Ⅰ real-time fluorescent quantitative PCR for detection of porcine reproductive and respiratory syndrome virus and porcine circovirus type 4**. China, *Chinese Journal of Veterinary Science*. 2022, 42(11):2158-2163.

99. Liu B, Zhao CQ, Liu LM, Sha WL, Dong WL, Li GJ. **Establishment and preliminary application of a triplex RT-qPCR for detection of porcine parvovirus, pseudorabies virus and porcine reproductive and respiratory syndrome virus**. China, *Journal of Jilin Agricultural Science and Technology University*. 2022, 31(02):1-5+25.

100. Xia W, Chen Y, Ding X, Liu X, Lu H, Guo C, Zhang H, Wu Z, Huang J, Fan Z *et al*: **Rapid and Visual Detection of Type 2 Porcine Reproductive and Respiratory Syndrome Virus by Real-Time Fluorescence-Based Reverse Transcription Recombinase-Aided Amplification**. *Viruses* 2022, **14**(11).

101. Yu XY, Li TZ, Li SG, Liu XJ, Li ZW, Shen ZQ, Xiao YQ. **Establishment of a duplex fluorescent PCR for detection of porcine reproductive and respiratory syndrome virus and Mycoplasma hyopneumoniae**. China, *Chinese Journal of Veterinary Medicine*. 2023, 59(06):60-64.

102. Li X. **Establishment and application of a quadruple real-time fluorescent quantitative PCR for detection of porcine respiratory viruses**. China, *Master's thesis*. 2023.

103. Gong BJ. **Establishment and application of a real-time fluorescent RT-PCR for detection of different genotypes of PRRSV**. China, *Master's thesis*. 2023.

104. Ruan S, Ren W, Yu B, Yu X, Wu H, Li W, Jiang Y, He Q: **Development and Implementation of a Quadruple RT-qPCR Method for the Identification of Porcine Reproductive and Respiratory Syndrome Virus Strains**. *Viruses* 2023, **15**(9).

105. Cao XZ, Zhou QF, Wang LX, Lin LM, Yu XG, Li W, Li QH. **Establishment of a fluorescent quantitative RT-PCR for detection of NADC34-like PRRSV**. China, *Shanghai Journal of Animal Husbandry and Veterinary Medicine*. 2023(05):7-13+20.

106. Chen L. **Establishment and application of a one-step RT-PCR for detection of porcine reproductive and respiratory syndrome virus**. China, *Fujian Journal of Animal Husbandry and Veterinary Medicine*. 2023, 45(04):27-30.

107. Wang YP, Li YH, Cao DQ, Huang RY, Li ZW, Pu TC, Wang H, Zeng H, Yang XW, Liu X, et al. **Establishment and clinical validation of a multiplex RT-PCR for detection of classical swine fever, porcine reproductive and respiratory syndrome and Japanese encephalitis**. China, *Guizhou Journal of Animal Husbandry and Veterinary Medicine*. 2023, 47(01):52-56.

108. Zhang YX, Wu Y, Zhang W, Tian YK, Huang Y, Zeng ZY, Wu XX, Wang B. **Establishment of a quadruple PCR for detection of porcine reproductive and respiratory syndrome virus, pseudorabies virus, porcine circovirus type 2 and swine influenza virus**. China, *Progress in Veterinary Medicine*. 2023, 44(08):22-27.

109. Men XK, Sun TT, Luo HY, Li S, Zhu JR, Jiang JJ, Sun Q, Shen W, Zhang CX, Chen C, et al. **Establishment of a multiplex PCR for detection of CSFV, ASFV and PRRSV**. China, *Chinese Journal of Animal Infectious Diseases*:1-8.

110. Li B, Zhao S, Zhou YN, Zhao W, Jiang JX, He Y, Guo X, Lu BX, Lin CH, Qin YB, et al. **Establishment and application of a multiplex PCR for detection of JEV, PRRSV, PPV and PRV**. China, *Progress in Veterinary Medicine*. 2023, 44(03):48-53.

111. Zhou Q, Yu KS, Lin QQ, Li WZ, Li WX, Yu Q, Wang QX, Chen L, Wang YQ. **A duplex fluorescent quantitative PCR method for detection of porcine reproductive and respiratory syndrome virus and differentiation of NADC30-like strains**. China, *Chinese Veterinary Science*. 2023, 53(04):462-467.

112. Zhao S, Chen ZW, Xiao T, He Y, Zhang N, Qin GX, Lu BX, Duan ZH, Qin YB, Zhang SB, et al. **Establishment and preliminary application of a multiplex TaqMan fluorescent quantitative PCR for detection of ASFV and PRRSV**. China, *Chinese Veterinary Science*. 2023, 53(06):671-678.

113. Chen Y, Zhong XY, Wu CY, Ding X, Huang J, Wang HL, Fan ZJ, Sun J, Chen XY, Yu SP, et al. **Rapid detection of genotype 2 porcine reproductive and respiratory syndrome virus by reverse transcription-recombinase aided amplification (RT-RAA)**. China, *China Animal Health Inspection*. 2023, 40(01):121-127.

114. Chen SZ. **Establishment of an isothermal amplification detection method based on PRRSV ORF2a gene and construction of its N-glycosylation site mutants**. China, *Master's thesis*. 2023.

115. Zhou Q. **Application of microfluidic molecular diagnostic technology MAOPA in PRRS eradication**. China, *Master's thesis*. 2023.

116. Ji C, Zhou L, Chen Y, Fang X, Liu Y, Du M, Lu X, Li Q, Wang H, Sun Y *et al*: **Microfluidic-LAMP chip for the point-of-care detection of gene-deleted and wild-type African swine fever viruses and other four swine pathogens**. *Front Vet Sci* 2023, **10**:1116352.

117. Tran DH, Lam NAN, Tran HT, Pham TNM, Trinh TBN, Nguyen VT, Le VP, Phung HTT: **Instrument-free, visual and direct detection of porcine reproductive and respiratory syndrome viruses in resource-limited settings**. *ACTA VIROLOGICA* 2023, **67**(1):69-+.

118. Li X, Yang L, Liu GL, Wang WX, Liu HL, Cao ZX, Zhang Y. **Establishment and application of a quadruple real-time fluorescent quantitative PCR for detection of PRRSV, CSFV, PRV and PCV2**. China, *Progress in Veterinary Medicine*. 2024, 45(06):7-14.

119. Sun TT. **Establishment of a duplex fluorescent quantitative PCR for detection of PRRSV and PCV2 and epidemiological analysis**. China, *Master's thesis*. 2024..

120. Feng Z, Shi K, Yin Y, Shi Y, Feng S, Long F, Wei Z, Si H: **A Quadruplex RT-qPCR for the Detection of African Swine Fever Virus, Classical Swine Fever Virus, Porcine Reproductive and Respiratory Syndrome Virus, and Porcine Pseudorabies Virus**. *Animals (Basel)* 2024, **14**(23).

121. Ma Y, Shi K, Chen Z, Shi Y, Zhou Q, Mo S, Wei H, Hu L, Mo M: **Simultaneous Detection of Porcine Respiratory Coronavirus, Porcine Reproductive and Respiratory Syndrome Virus, Swine Influenza Virus, and Pseudorabies Virus via Quadruplex One-Step RT-qPCR**. *Pathogens* 2024, **13**(4).

122. Tao C, Zhu X, Huang Y, Yuan W, Wang Z, Zhu H, Jia H: **Development of a Multiplex RT-qPCR Method for the Identification and Lineage Typing of Porcine Reproductive and Respiratory Syndrome Virus**. *Int J Mol Sci* 2024, **25**(23).

123. Wang G, Zhu H, Zhan C, Chen P, Wu B, Peng Z, Qian P, Cheng G: **Establishment and Application of a Quadruplex Real-Time Reverse-Transcription Polymerase Chain Reaction Assay for Differentiation of Porcine Reproductive and Respiratory Syndrome Virus, Porcine Circovirus Type 2, Porcine Circovirus Type 3, and Streptococcus suis**. *Microorganisms* 2024, **12**(3).

124. Xu Y, Yu LZ, Lin S, Ren TW, Wang H, Zeng H, Guo JN, Guo JF, Huang CQ, Ouyang K, et al. **Establishment and application of a multiplex PCR for detection of H1-SIV, H3-SIV, NA-PRRSV and EU-PRRSV**. China, *Chinese Journal of Animal Infectious Diseases*. 2024, 32(01):135-141.

125. Liu Y, Yan RQ, Wang DF, Yang HB, Zhao MX, Song D, Zhao XL, Xie CH, Wang SJ, Ma ZY, et al. **Establishment and preliminary application of a duplex real-time fluorescent quantitative PCR for detection of African swine fever virus and highly pathogenic porcine reproductive and respiratory syndrome virus**. China, *Chinese Journal of Animal Infectious Diseases*. 2024, 32(02):118-130.

126. Yang C, Li WJ, Zhang XF, Cheng N, Liu Y, Wang KY, Shao X, Li SY, Sun YF. **Establishment and application of a multiplex PCR for detection of CSFV, PCV2 and PRRSV in boar semen**. China, *Progress in Veterinary Medicine*. 2024, 45(09):77-80.

127. An LL, Luo X, Yang XY, Hu XY, Zhao YQ. **Establishment of a real-time fluorescent quantitative PCR for detection of porcine reproductive and respiratory syndrome virus ORF7 gene**. China, *Jiangsu Journal of Agricultural Sciences*. 2024, 40(09):1681-1688.

128. Yu N, Ma JM, Zhang ZW, Huang CY, Fan YX, Zheng JX, Zhang Y, Liu GL, Cao ZX. **Establishment of an RT-RAA assay for porcine reproductive and respiratory syndrome virus based on ORF7 gene**. China, *Progress in Veterinary Medicine*. 2024, 45(08):117-119.

129. Yu N. **Establishment of an RT-RAA detection method for porcine reproductive and respiratory syndrome virus and screening of N protein hybridoma cells**. China, *Master's thesis*. 2024.

130. Chen R, Yu XL, Gao XB, Xue CY, Song CX, Li Y, Cao YC: **Bead-based suspension array for simultaneous differential detection of five major swine viruses**. *Appl Microbiol Biotechnol* 2015, **99**(2):919-928.

131. Gou H, Deng J, Pei J, Wang J, Liu W, Zhao M, Chen J: **Rapid and sensitive detection of type II porcine reproductive and respiratory syndrome virus by reverse transcription loop-mediated isothermal amplification combined with a vertical flow visualization strip**. *J Virol Methods* 2014, **209**:86-94.

132. Liu SL. **Establishment of multiplex PCR and combined gene chip detection methods for five porcine reproductive disorder viruses**. China, *Master's thesis*. 2018.

133. Wu XL, Xiao L, Lin H, Yang ZX, Yao XP, Wang Y, Zhang PF, Jiang RJ. **Establishment and preliminary application of a detection method for seven swine viruses based on QIAxcel capillary gel electrophoresis analysis system**. China, *Chinese Journal of Preventive Veterinary Medicine*. 2019, 41(08):824-829.

134. Liu SL, Liu LL, Lyu YJ, Wu FS, Li WG. **Research on multiplex PCR combined with gene chip technology for detection of five porcine reproductive disorder viruses**. China, *China Animal Husbandry & Veterinary Medicine*. 2019, 46(05):1532-1540.

135. Zeng YY, Li CY, Ren YP, Zhang HR. **Establishment and preliminary application of RPA-LFD diagnostic method for highly pathogenic porcine reproductive and respiratory syndrome**. China, *Journal of Southwest Minzu University (Natural Science Edition)*. 2022, 48(04):379-385.

136. Li WS, Li MH, Zhang HL, Li C, Xu H, Fu J, Gong BJ, Guo ZY, Liu JB, Peng JM, et al. **Development and preliminary application of an immunochromatographic strip for North American genotype porcine reproductive and respiratory syndrome virus**. China, *Chinese Journal of Preventive Veterinary Medicine*. 2022, 44(08):842-849.

137. Bi JY. **Construction of armored RNA quality control material for PRRSV and establishment of RPA detection method**. China, *Master's thesis*. 2023.

138. Chen SZ, Ma JM, Huang CY, Fan YX, Zheng JX, Liu GL, Cao ZX. **Establishment of an RT-RAA-LF detection method based on ORF2a gene of porcine reproductive and respiratory syndrome virus**. China, *Chinese Veterinary Science*. 2023, 53(10):1249-1253.

139. Zheng H. **Establishment of a fluorescent microsphere test strip for diagnosis of porcine reproductive and respiratory syndrome**. China, *Master's thesis*. 2023.

140. Wu XL, Xiao L, Yang M, Xie L, An W, Guo Y, Zhang Q, Wang Y, Lin H. **Establishment and application of a rapid liquid chip detection method for seven porcine reproductive disorders**. China, *Journal of Agricultural Biotechnology*. 2023, 31(01):203-212.

141. Yu JX, Wei SS, Qin SM, Wu JM, Yang LH, Chen FL, Xu LS, Qin SY, Hua J, Wei J, et al. **Establishment of a rapid detection method for porcine reproductive and respiratory syndrome virus based on CRISPR/Cas12a-RT-RAA**. China, *China Animal Husbandry & Veterinary Medicine*. 2024, 51(08):3237-3246.

142. Zhang ZW, Ma JM, Huang CY, Yu N, Zheng JX, Zhang Y, Liu GL, Cao ZX. **Establishment of an RT-RAA-LF detection method based on ORF7 gene of porcine reproductive and respiratory syndrome virus**. China, *Chinese Journal of Animal Infectious Diseases*:1-7.
